# CFTR in alveolar type 1 cells contributes to lung liquid secretion and host defense

**DOI:** 10.1172/jci.insight.194121

**Published:** 2026-08-11

**Authors:** Sayahi Suthakaran, Sonya Homami, Deebly Chavez, Stephanie Tang, Sarah K.L. Moore, Chaya Sussman, Jimmy Zhang, Clemente J. Britto, Alice Prince, Alison J. May, Jaymin J. Kathiriya, Jaime L. Hook

**Affiliations:** 1 Division of Pulmonary, Critical Care, and Sleep Medicine, Department of Medicine, and; 2 Graduate School of Biomedical Sciences, Icahn School of Medicine at Mount Sinai, New York, New York, USA.; 3 Division of Pulmonary, Critical Care, and Sleep Medicine, Department of Medicine, Yale University School of Medicine, New Haven, Connecticut, USA.; 4 Department of Pediatrics, College of Physicians and Surgeons, Columbia University Medical Center, New York, New York, USA.; 5 Department of Stem Cell Biology and Regenerative Medicine,; 6 Department of Otolaryngology,; 7 Department of Microbiology, and; 8 Global Health and Emerging Pathogens Institute, Icahn School of Medicine at Mount Sinai, New York, New York, USA.

**Keywords:** Cell biology, Infectious disease, Pulmonology, Bacterial infections, Chloride channels, Epithelial transport of ions and water

## Abstract

CFTR in the lung epithelium contributes to the secretion of a surface liquid layer that is essential to lung homeostasis and defense. The understanding of how liquid is secreted in the lung is derived largely from studies of the airway epithelium. Comparatively little is known about liquid secretion mechanisms in the alveolar epithelium, including its cellular source. To define which cell type drives alveolar liquid secretion, we generated transgenic mice that expressed a *Cftr-*null allele in alveolar type 1 (AT1) cells, type 2 (AT2) cells, or both, then viewed liquid secretion in live alveoli using confocal microscopy of isolated, perfused lungs. Our findings show that liquid secretion was blocked in alveoli of all 3 transgenic mice, indicating that both AT1 and AT2 cells contribute to alveolar liquid secretion. *Cftr-*null expression in AT1 cells also blocked the secretion-mediated clearance of small particle and bacterial clusters from alveolar walls, indicating that AT1 cell CFTR contributes to alveolar defense. Together, these findings show alveolar liquid secretion depends on both AT1 and AT2 cell CFTR, and that CFTR in AT1 cells — a cell type not traditionally considered in liquid secretion mechanisms or CFTR-related lung diseases — contributes to lung liquid dynamics and host defense.

## Abstract 2

This work shows CFTR function in AT1 cells – a cell considered immaterial to CFTR-related diseases – is critical to CFTR-mediated lung liquid secretion and defense.

## Introduction

The lung’s air-facing epithelial surface is lined by an aqueous liquid layer that prevents epithelial cell desiccation and contributes to host defense. Loss of this liquid layer has a role in the pathogenesis of diverse lung diseases including cystic fibrosis (CF) (1, 2), chronic obstructive pulmonary disease (COPD) (3, 4), and lung infection (5–7). Much of the understanding of liquid secretion mechanisms in the lung is derived from studies of liquid transport in the airway epithelium. Although the alveolar epithelium also secretes liquid and comprises over 95% of the lung’s air-facing surface area (5, 8, 9), comparatively little is known about liquid secretion in alveoli, including its cellular source.

Electron microscopic studies of rat lungs show the alveolar epithelium is lined by a continuous layer of surface liquid (10), and experiments in live alveoli of isolated, perfused lungs provide evidence that the liquid is secreted by the alveolar epithelium in a CFTR-dependent manner (5, 6, 11). CFTR is expressed in both alveolar type 1 (AT1) and type 2 (AT2) cells in adult human lungs (12, 13), raising the possibility that CFTR in both cell types contributes to liquid secretion in alveoli at homeostasis. In vitro studies support the possibility that both cell types contribute, since reports of cultured and freshly isolated alveolar epithelial cells show that both AT1 (14–18) and AT2 (19–25) cells transport Na^+^, Cl^–^, and liquid. However, the extent to which CFTR in each cell type contributes to alveolar liquid secretion in vivo remains an open question. Whether CFTR in AT1 cells contributes is particularly unclear, since the extensive cell surface area that makes AT1 cells likely to contribute to alveolar liquid secretion has also made AT1 cells traditionally challenging to isolate and study.

In this work, we used recently developed genetic tools to quantify the contribution of AT1 and AT2 cell CFTR to alveolar liquid secretion. We generated transgenic mice that express a *Cftr-*null allele in AT1 cells, AT2 cells, or both cell types, then used confocal microscopy to view liquid secretion in alveoli of live, perfused lungs. Our findings show that alveolar liquid secretion was blocked in all 3 transgenic mice, indicating that CFTR in both AT1 and AT2 cells contributes to alveolar liquid secretion under baseline conditions. In addition, *Cftr-*null expression in AT1 cells inhibited the secretion-mediated displacement of small clusters of particles and bacteria from alveolar walls, indicating that AT1 cell CFTR defends against particle and bacterial stabilization in alveoli. Together, these data provide the first evidence, of which we are aware, that CFTR in both AT1 and AT2 cells contributes to alveolar liquid secretion. We propose that AT1 cells are important for lung liquid secretion and host defense.

## Results

### AT1 and AT2 cell–specific CFTR^null^ expression in transgenic mice.

We bred widely used transgenic mouse strains to generate mice in which a *Cftr-*null allele and GFP expression were tamoxifen induced in adulthood in an AT1 or AT2 cell–specific manner. Thus, AT2-CFTR^null^-GFP mice carried *Sftpc-CreER^T2^* (26), *Cftr^fl10/fl10^* (27), and *ROSA^mT/mG^*, a reporter allele that confers membrane-targeted TdTomato expression prior to Cre-mediated excision, and GFP expression afterward (28). Likewise, AT1-CFTR^null^-GFP mice carried *Ager-CreER^T2^* (29), *Cftr^fl10/fl10^*, and *ROSA^mT/mG^*; AT2-AT1-CFTR^null^-GFP mice carried *Sftpc-CreER^T2^*, *Ager-CreER^T2^*, *Cftr^fl10/fl10^*, and *ROSA^mT/mG^*; and LMC (LMC-TdTomato) mice carried *Cftr^fl10/fl10^* and *ROSA^mT/mG^*.

**Figure 1:**
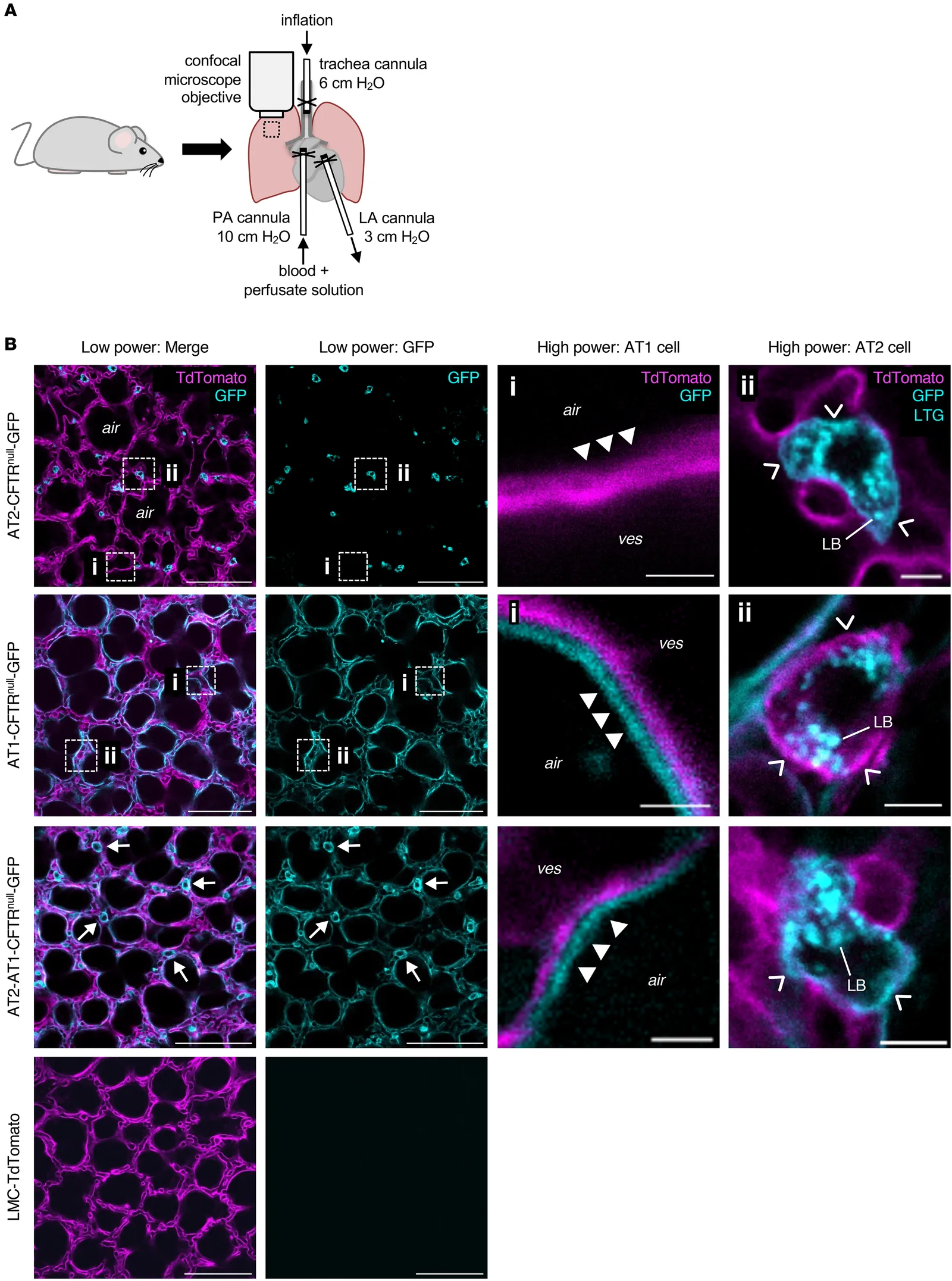
Alveolar epithelial cell type-specificity of GFP expression. (**A**) The illustration shows the experimental design of confocal imaging studies of live, intact, perfused lungs of transgenic mice. (**B**) Confocal images show plasma membrane GFP (cyan) and TdTomato (magenta) fluorescence in live lungs of mice of the indicated genotype. Dashed squares show locations of the high power views shown in the third and fourth columns. Closed arrowheads point out AT1 cell fluorescence, which is GFP^+^ (cyan) and distinct from neighboring TdTomato^+^ (magenta) endothelial cells only in AT1-CFTR^null^-GFP and AT2-AT1-CFTR^null^-GFP mice. Open arrowheads show plasma membrane fluorescence of AT2 cells, which is GFP^+^ (cyan) only in AT2-CFTR^null^-GFP and AT2-AT1-CFTR^null^-GFP mice. In the fourth column, cytosolic fluorescence of lysotracker green (LTG) delineates lamellar bodies (LB) and confirms AT2 cell identity. In the third row, arrows show example AT2 cells in an AT2-AT1-CFTR^null^-GFP mouse. Air, airspace; ves, microvessel lumen. Scale bars: 100 (first and second columns), 3 (third columns), and 5 (fourth columns) μm. Images were replicated in lungs of 3 mice per genotype. Note, in the fourth column, both LTG and GFP are pseudocolored cyan.

We used our established methods (6, 30) to view live alveoli of intact, perfused lungs of AT2-CFTR^null^-GFP mice by real-time confocal microscopy (Figure 1A). GFP fluorescence was evident in plasma membranes of rounded cells that were located primarily at septal junctions (Figure 1B), consistent with AT2 cell morphology and location (31, 32). To test AT2 cell function, we used alveolar micropuncture to microinstill alveolar airspaces with LysoTracker dye, which is taken up by AT2 cells and localizes to AT2 cell lamellar bodies. Hyperinflation-induced LysoTracker fluorescence loss signals surfactant secretion (33). Our findings show lung hyperinflation caused time-dependent loss of LysoTracker fluorescence in GFP^+^ cells (Supplemental Figure 1, A and B; supplemental material available online with this article; https://doi.org/10.1172/jci.insight.194121DS1), affirming that GFP^+^ cells in AT2-CFTR^null^-GFP mice function as AT2 cells. To quantify *Cftr* deletion efficiency, we isolated AT2 cells (Supplemental Figure 2, A–C) and performed qPCR of *Cftr* RNA using our established methods (34, 35). AT2 cells of an AT2-CFTR^null^-GFP mouse had 100-fold reduction of *Cftr* RNA compared with AT2 cells of an LMC-TdTomato mouse (Supplemental Tables 1 and 2). Taking these findings together, we conclude that AT2 cells of AT2-CFTR^null^-GFP mice are *Cftr* deficient and marked by GFP fluorescence.

By contrast, GFP fluorescence in live lungs of AT1-CFTR^null^-GFP mice was evident in thin cells that line alveolar walls (Figure 1B), consistent with AT1 cell morphology and location. AT2 cells of AT1-CFTR^null^-GFP mice had abundant *Cftr* RNA (Supplemental Figure 2D and Supplemental Table 1). Taking these data together, we interpret that CFTR^null^ expression in AT1-CFTR^null^-GFP mice is AT1 cell specific.

In AT2-AT1-CFTR^null^-GFP mice, GFP fluorescence was evident in plasma membranes of both AT2 and AT1 cells (Figure 1B), and AT2 cell qPCR showed more than 100-fold reduction of *Cftr* RNA (Supplemental Figure 2E and Supplemental Table 1). There was no GFP fluorescence in alveoli of LMC-TdTomato mice (Figure 1B). We interpret that AT2-AT1-CFTR^null^-GFP mice are *Cftr* deficient in both AT2 and AT1 cells, while LMC-TdTomato mice are *Cftr* deficient in neither cell type.

### CFTR^null^ expression is heterogeneous across alveoli of Ager-CreER^T2^–expressing mice.

**Figure 2:**
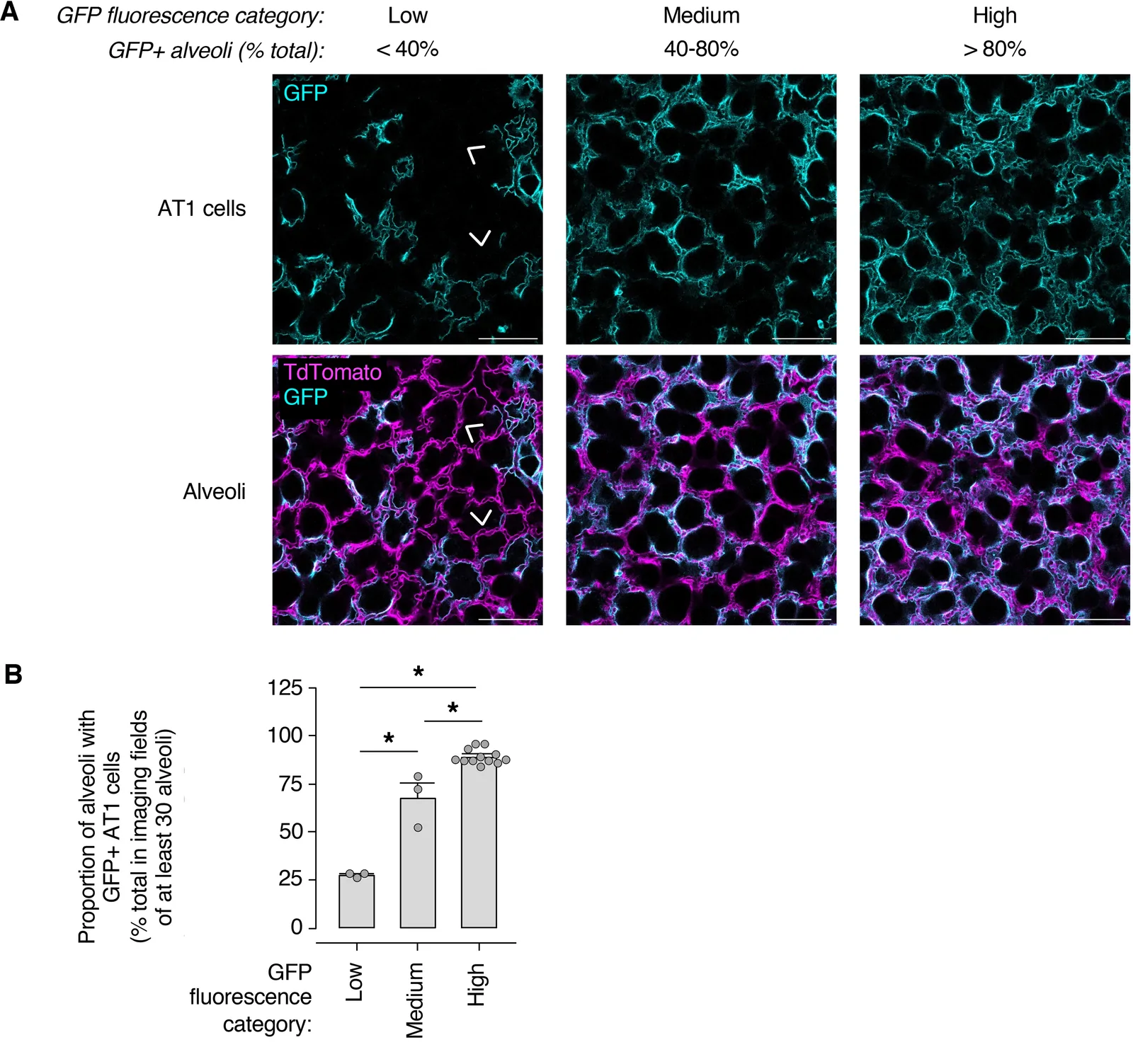
Cre recombination is spatially heterogeneous across alveoli of AT1-CFTR^null^-GFP mice. (**A** and **B**) Confocal images (**A**) and group data (**B**) show GFP fluorescence (cyan) across imaging fields of at least 30 alveoli in live, perfused lungs of AT1-CFTR^null^-GFP mice. In the images in **A**, cyan fluorescence marks Cre-expressing AT1 cells. Note, Cre expression is heterogeneous across alveoli, since some AT1 cells expressed TdTomato, but not GFP, as evident by TdTomato (magenta) fluorescence in GFP-free alveoli (examples: arrowheads). The group data in **B** quantify the heterogeneity by indicating the proportion of alveoli with GFP^+^ cells as a percentage of the total number of alveoli per imaging field. Circles in **B** indicate *n* and each represent 1 imaging field of at least 30 alveoli. Imaging fields were generated in lungs of 3 mice but presented according to their GFP fluorescence category. Data are shown as mean ± SEM; *P < 0.05 by ANOVA with post hoc Tukey testing. Scale bars: 100 μm.

Tamoxifen-treated *Ager-CreER^T2^*–expressing mice show Cre recombination efficiency that varies across lung alveoli (29), leading to a heterogeneous distribution of Cre expression in which AT1 cells of some alveoli express Cre, while AT1 cells of other alveoli do not. In line with the published data, our images show that AT1 cell GFP fluorescence was heterogeneous across imaging fields of alveoli in lungs of AT1-CFTR^null^-GFP mice (Figure 2, A and B), while TdTomato fluorescence was evident in all alveoli (Figure 2A). AT2-AT1-CFTR^null^-GFP showed similar heterogeneity of GFP expression (data not shown). We categorized the proportion of GFP-expressing AT1 cells in imaging fields of AT1-CFTR^null^-GFP mice as Low, Medium, or High if GFP expression was evident in less than 40%, 40%–80%, or more than 80%, respectively, of alveoli in the imaging field (Figure 2, A and B). We interpreted the GFP fluorescence categories to indicate sparse, moderate, and extensive CFTR^null^ expression, respectively, across imaging fields of alveoli.

### CFTR in AT1 cells and AT2 cells contributes to alveolar liquid secretion.

We next aimed to define the effect of AT1 and AT2 cell CFTR^null^ expression, and its heterogeneity, on alveolar function. To do this, we applied a technique that has been used previously to identify liquid secretion and absorption in alveoli of live, perfused lungs using upright confocal microscopy (5, 6). Briefly, the experiments are carried out by microinstilling alveolar airspaces with fluorophore-conjugated dextran in aqueous solution. The dextran-containing solution is visible in alveoli by confocal microscopy as a juxta-epithelial layer that pools at alveolar niches, where septa converge (5, 6). Since the fluorescence intensity of the dextran-containing solutions varies with dextran concentration (6), the solutions lose fluorescence in airspaces as they become diluted by nonfluorescent alveolar wall liquid (AWL) secreted by the alveolar epithelium (5, 6). Conversely, the solutions gain fluorescence when the epithelium absorbs liquid from airspaces (5, 6). Since secreted AWL flows toward airways and does not accumulate in alveoli (5), the widths of the dextran-containing solution pools are unchanged over time when AWL is secreted (5, 6). However, they diminish over time when airspace liquid is absorbed (5, 6).

Using this approach, we and others have reported alveoli of intact, perfused mouse lungs secrete AWL in a CFTR-dependent manner under baseline conditions (5, 6). A recent study from our group affirms CFTR dependence and adds that the direction of alveolar liquid transport depends on lung perfusion, since alveoli of lungs perfused at or near physiological levels secrete AWL, while alveoli of lungs in which perfusion is majorly reduced or halted absorb airspace liquid (36). Microvessel-to-airspace osmotic gradients do not seem to determine the direction of alveolar liquid transport (36). Because fluid leak from microvessels into airspaces in perfused lungs could dilute the dextran-containing solutions, we ruled out alveolar leak by showing that airspace dextran fluorescence decreased, then increased in the same alveoli, under conditions of continuous perfusion, in response to changes of pulmonary artery pressure (36). Since airspace dextran fluorescence could not increase in alveoli with barrier leak, these data align with reports indicating alveolar barrier function is intact in isolated, perfused lungs (37, 38). Finally, we have used airspace instillation of fluorophore-tagged antibodies to show alveolar macrophages are present in airspaces of isolated, perfused lungs (36), indicating that expected nonepithelial cell types are present in our preparation. Neutrophils are absent in airspaces under baseline conditions but recruited after intranasal instillation of *S*. *aureus* (6).

**Figure 3:**
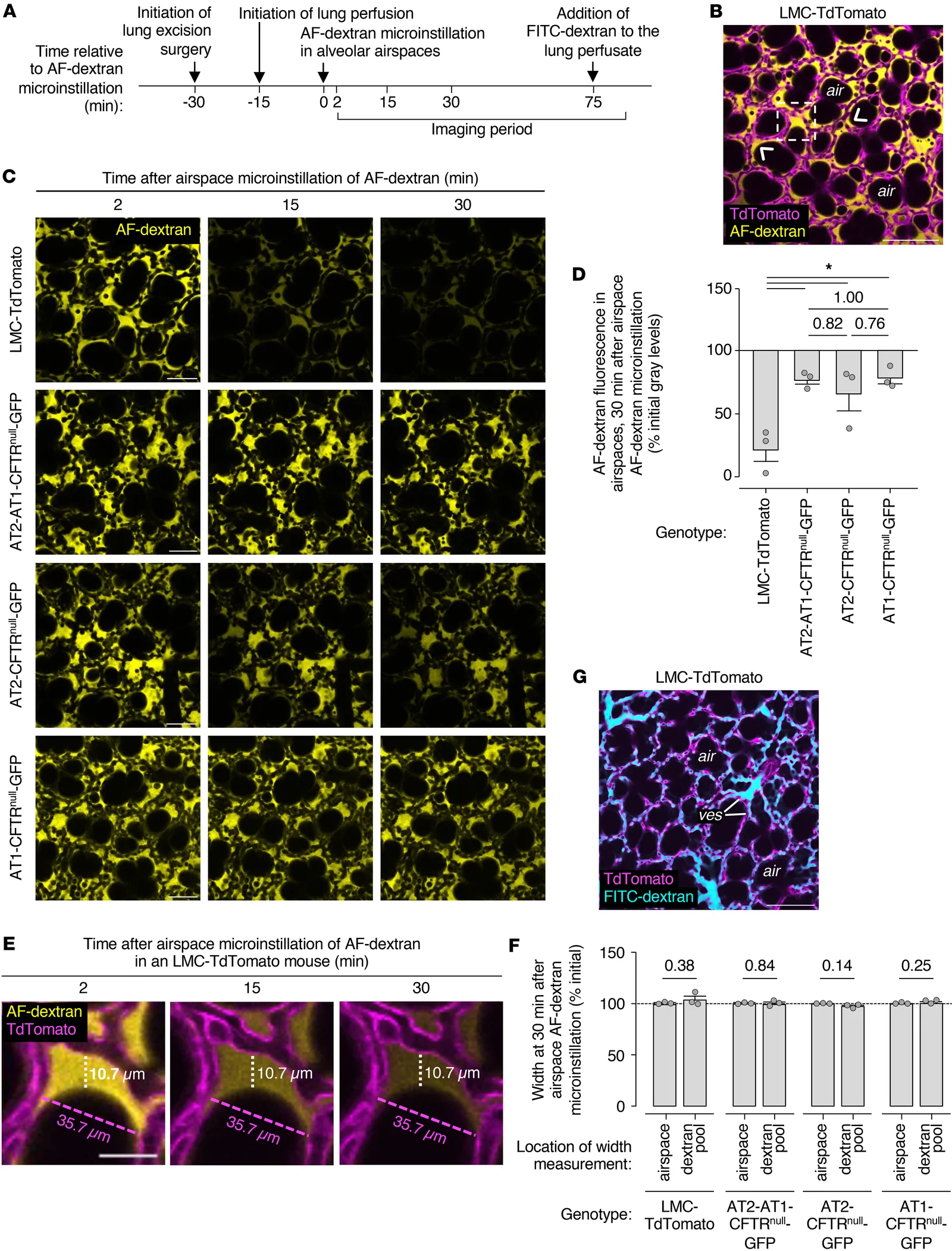
Real-time confocal imaging of liquid secretion in live alveoli. (**A**–**G**) Illustration in **A** shows the design of confocal imaging studies of liquid transport in live alveoli of intact, perfused lungs of transgenic mice of the genotypes indicated in **B**–**G**. As indicated in **A**, we microinstilled alveolar airspaces with Alexa Fluor 647–conjugated dextran (AF-dextran, yellow, 10 kDa, 10 mg/mL) to carry out the determinations of liquid transport shown in **B**–**F**. At the conclusion of each experiment, we added fluorescein isothiocyanate-conjugated dextran (FITC-dextran, cyan, 20 kDa, 5 mg/mL) to the lung perfusate solution to define alveolar barrier properties. The confocal image in **B** shows AF-dextran fluorescence in airspaces of alveoli (magenta) of a TdTomato-expressing LMC-TdTomato mouse at 2 minutes after AF-dextran microinstillation. Air, example airspace. Arrowheads point out example AF-dextran pools at alveolar niches. Images and group data in **C** and **D** show change of AF-dextran fluorescence over time. Fluorescence of alveolar walls is not shown. In **D**, circles indicate *n* and each represent one mouse in which mean fluorescence change was quantified in imaging fields of at least 30 alveoli; data are shown as mean ± SEM; *P < 0.05 by ANOVA with post hoc Tukey testing. High power views in **E** show widths of the example AF-dextran pool (white dashed lines and text) and airspace (magenta dashed lines and text) indicated by the dashed square in **B**. Group data in **F** show pool and airspace widths over time. In **F**, circles and bars are as in **D**; *P* is as indicated by 2-tailed *t* test. The confocal image in **G** shows fluorescence of FITC-dextran added to the lung perfusate solution. Images in **B** and **G** represent images generated in 3 mice each of LMC-TdTomato, AT2-AT1-CFTR^null^-GFP, AT2-CFTR^null^-GFP, and AT1-CFTR^null^-GFP mouse groups. Scale bars: 100 (**B** and **G**), 50 (**C**), 20 (**E**) μm.

We now applied these methods to test the hypothesis that AT1 and AT2 cell CFTR^null^ expression inhibits AWL secretion. We microinstilled alveolar airspaces of LMC-TdTomato, AT2-AT1-CFTR^null^-GFP, AT2-CFTR^null^-GFP, and AT1-CFTR^null^-GFP mice with Alexa Fluor 647–conjugated dextran (AF-dextran; 10 kDa; 10 mg/mL) (Figure 3A). We used AF-dextran because its fluorescence intensity varies with concentration in aqueous solution (Supplemental Figure 3, A and B), and its excitation and emission spectra are distinct from those of GFP and TdTomato. The contents of the airspace instillate and lung perfusate solutions for our experiments are shown in Supplemental Table 3. In alveoli of AT1-CFTR^null^-GFP and AT2-AT1-CFTR^null^-GFP mice, we restricted our analysis to imaging fields that had High-GFP AT1 cell fluorescence, in order to avoid confounding the experiments with alveoli that lacked CFTR^null^ expression.

In alveoli of LMC-TdTomato mice, microinstilled AF-dextran lined alveolar walls and pooled at alveolar niches within minutes of microinstillation (Figure 3B), as expected. AF-dextran fluorescence subsequently decreased in a time-dependent manner (Figure 3, C and D), suggesting the alveolar epithelium secreted AWL. The widths of AF-dextran–containing liquid pools in alveoli were stable over time (Figure 3, E and F), also as expected, indicating that the volume of AF-dextran–containing liquid in airspaces did not increase during the imaging period. Stable airspace diameter during the same imaging period (Figure 3, E and F) indicates our measurements of AF-dextran pool width were not confounded by changes of alveolar morphology. To rule out the possibility that AF-dextran fluorescence loss was caused by leak of microvascular fluid into airspaces, we added fluorescein isothiocyanate–tagged (FITC-tagged) dextran (FITC-dextran, 20 kDa, 5 mg/mL) to the lung perfusate solution (Figure 3A), as we have done previously (6, 30). FITC-dextran fluorescence was visible in microvascular lumens but did not leak into alveolar airspaces (Figure 3G), indicating alveolar barrier function was intact during the imaging period. Together, these findings show alveoli secreted AWL in lungs of LMC-TdTomato mice.

By contrast, AF-dextran fluorescence loss in airspaces was blocked in alveoli of AT2-AT1-CFTR^null^-GFP mice (Figure 3, C and D). Widths of AF-dextran–containing liquid pools and airspaces in alveoli did not change over time (Figure 3, E and F), and FITC-dextran in the lung perfusate solution did not leak into airspaces. These findings show CFTR^null^ expression in both AT1 and AT2 cells inhibits AWL secretion, and they confirm reports indicating that AWL secretion depends on CFTR in the alveolar epithelium (5, 6). Notably, AWL secretion was not completely lost in alveoli of AT2-AT1-CFTR^null^-GFP mice (Figure 3, C and D). The residual secretion might reflect contributions to AWL secretion by non-CFTR Cl^–^ channels in the alveolar epithelium.

AF-dextran fluorescence loss was also blocked in alveoli of AT2-CFTR^null^-GFP mice and AT1-CFTR^null^-GFP mice (Figure 3, C and D). Widths of AF-dextran–containing liquid pools and airspaces in alveoli of both AT2-CFTR^null^-GFP and AT1-CFTR^null^-GFP mice were steady over time (Figure 3, E and F), and FITC-dextran that was added to the lung perfusate did not leak into airspaces. Together, these findings show CFTR^null^ expression in each of AT2 and AT1 cells inhibited liquid secretion in alveoli. We conclude that CFTR in both AT2 and AT1 cells contributes to AWL secretion.

**Figure 4:**
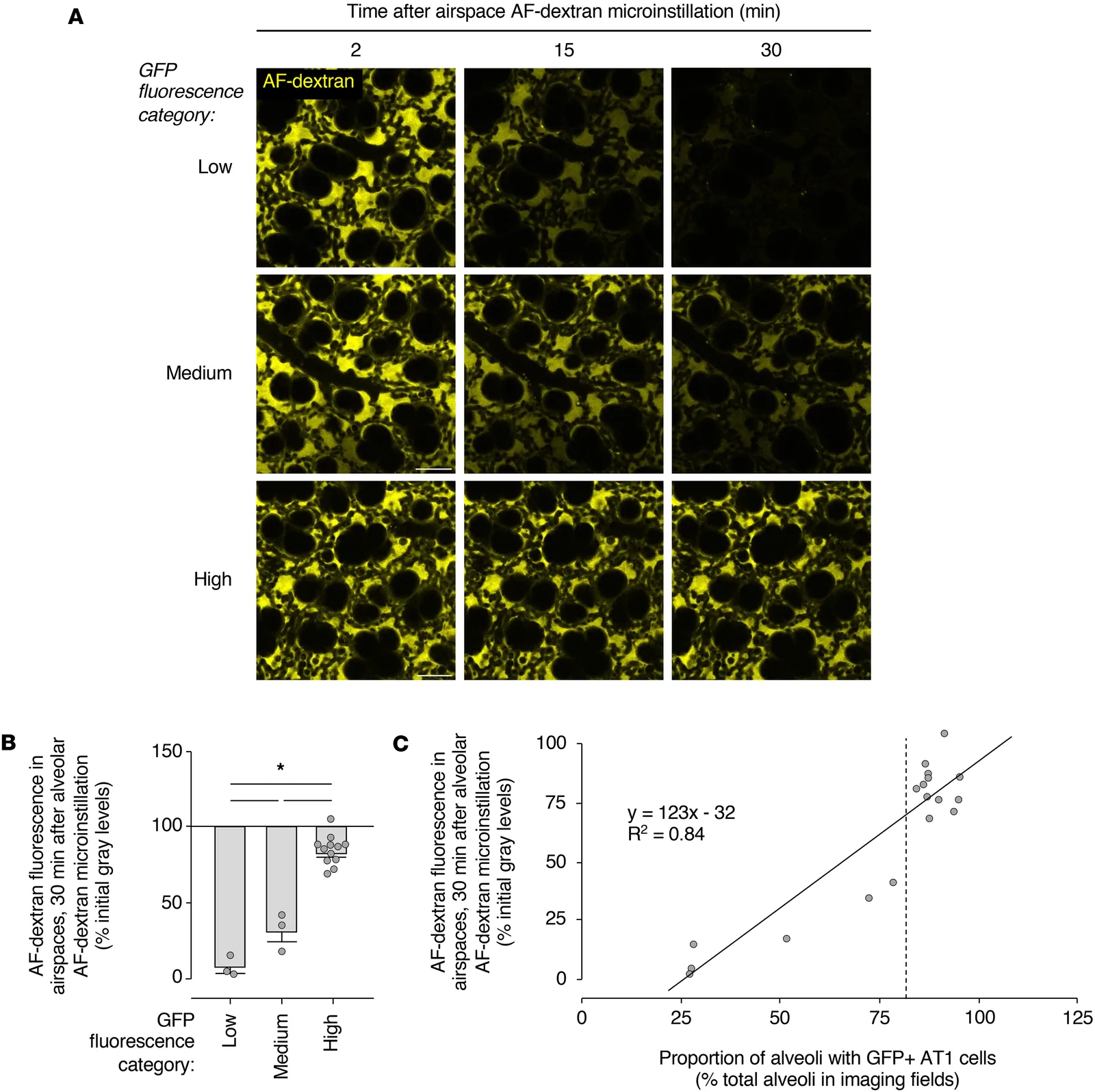
Dose-dependent relationship between AT1 cell *Cftr* deficiency and inhibition of AWL secretion in lungs of AT1-CFTR^null^-GFP mice. (**A**–**C**) Confocal images (**A**) and group data (**B** and **C**) show AF-dextran fluorescence (yellow*;* 10 kDa, 10 mg/mL) after microinstillation in alveolar airspaces of lungs of AT1-CFTR^null^-GFP mice. Experiments were carried out as indicated in Figure 3A. GFP fluorescence is not shown but was categorized in imaging fields of at least 30 alveoli as Low, Medium, and High as indicated in Figure 2. Images shown in the bottom row in **A** are reproduced from Figure 3C. In **B** and **C**, circles indicate *n* and each represent 1 trial of AF-dextran microinstillation in which mean fluorescence change was quantified in an imaging field of at least 30 alveoli. Trials were carried out in lungs of 3 mice but are presented according to the GFP fluorescence category (**B**) or the proportion of alveoli with GFP^+^ cells as a percentage of the total number of alveoli (**C**) in the imaging field. In **C**, the dashed line sets apart the 12 imaging fields in the High GFP fluorescence category, and the *s*olid line was calculated by linear regression. Data are shown as mean ± SEM; *P < 0.05 by ANOVA with post hoc Tukey testing. Scale bars: 50 μm.

Heterogeneous recombination efficiency across alveoli of tamoxifen-treated *Ager-CreER^T2^*–expressing mice (29) provides an opportunity to relate the magnitude of AT1 cell CFTR^null^ expression in imaging fields of alveoli to AWL secretion rates in lungs of AT1-CFTR^null^-GFP mice. To determine whether AWL secretion varies with the extent of AT1 cell CFTR^null^ expression, we reanalyzed the AF-dextran images that we generated in AT1-CFTR^null^-GFP mice by GFP expression category, as defined in Figure 2, rather than by mouse, as in Figure 3. Figure 4, A–C, shows the relationship between AF-dextran fluorescence change and AT1 cell GFP expression. Our findings show that, in Low-GFP imaging fields, airspace AF-dextran fluorescence decreased rapidly (Figure 4, A–C), indicating that sparse AT1 cell CFTR^null^ expression across alveoli had no effect on AWL secretion. However, AF-dextran fluorescence loss was slower in Medium-GFP imaging fields, and it was slower still in High-GFP imaging fields (Figure 4, A–C). These findings show the rate of AWL secretion depends on AT1 cell CFTR^null^ expression, such that imaged regions of alveoli that had the most CFTR^null^ expression had the least AWL secretion, and vice versa. We conclude that the extent of CFTR expression in AT1 cells determines AWL secretion. This direct, “dose-dependent” relationship between AT1 cell CFTR expression and AWL secretion strengthens our conclusion that AT1 cell CFTR is a driver of liquid secretion in alveoli.

We followed up by testing whether microvessel-to-airspace osmotic gradients promote AWL secretion and could have affected our interpretations. Our data show the AF-dextran–containing airspace solution that we used to generate Figure 3 and Figure 4 was hyperosmolar to the lung perfusate solution (Supplemental Figure 4A and Supplemental Table 3), raising the possibility that the microvessel-to-airspace osmotic gradient present in our preparation, which is favorable to AWL secretion, drove the AF-dextran fluorescence loss we observed in our experiments (Figures 3 and 4). To test this possibility, we quantified AF-dextran fluorescence changes in alveoli of WT mice that we microinstilled with modified AF-dextran solution that abolished the microvessel-to-airspace osmotic gradient by reducing the osmolality of the microinstillate solution (Supplemental Figure 4, A–D, and Supplemental Table 3). Our data show the airspace fluorescence of the modified AF-dextran solution decreased, while the AF-dextran pool width and alveolar airspace width were steady (Supplemental Figure 4, C and D). These findings show AWL secretion occurred in the absence of a prosecretion osmotic gradient between microvessels and airspaces and, thus, affirm findings included in a separate report from our group (36). We conclude that the direction of liquid transport in alveoli occurs independently of microvessel-to-airspace osmotic gradients. These data strengthen our conclusion that AT1 and AT2 cells contribute to AWL secretion at homeostasis.

### AT1 cell CFTR promotes particle clearance in alveoli.

**Figure 5:**
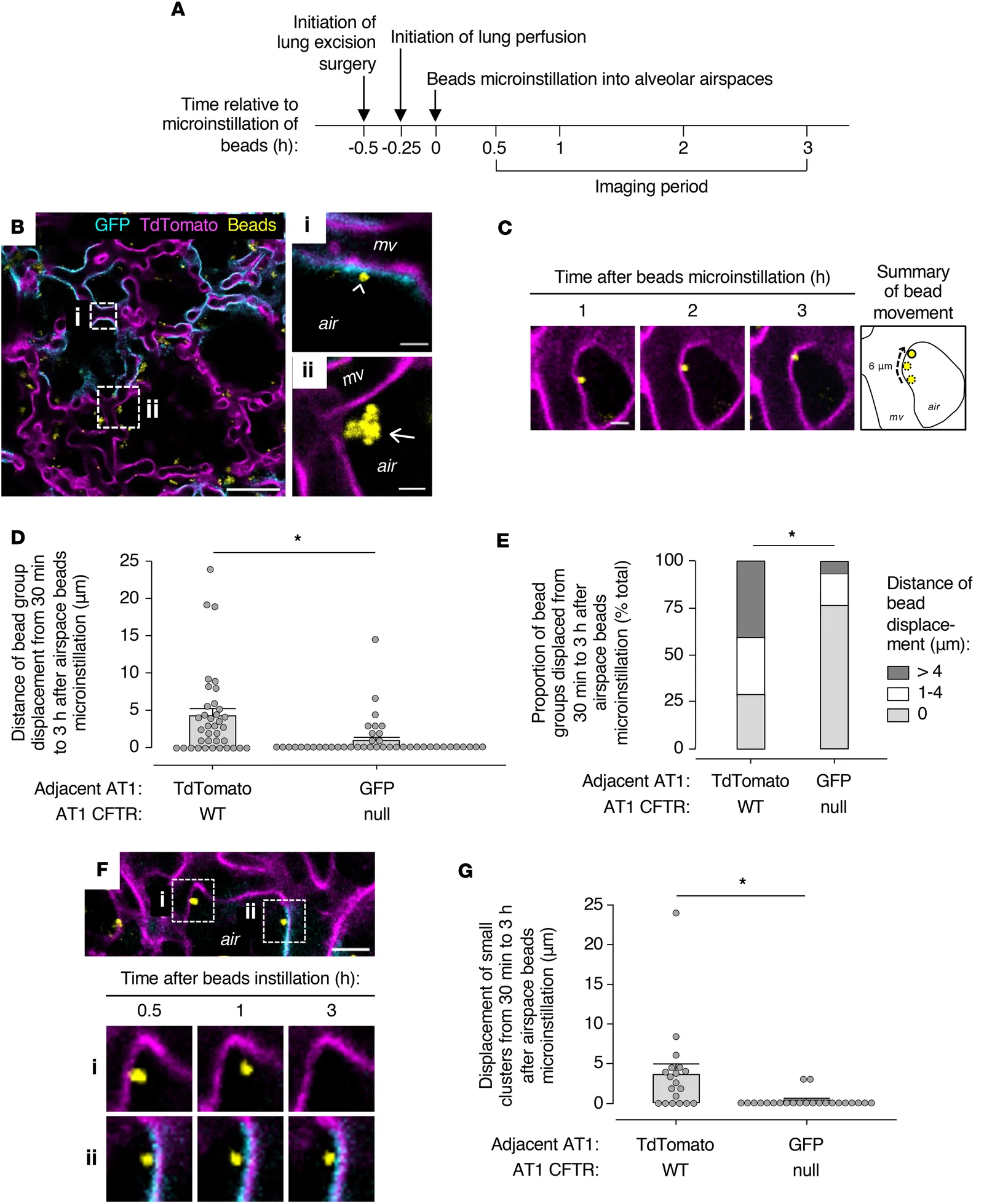
CFTR in AT1 cells promotes particle clearance in alveoli. (**A**–**G**) Illustration in **A** shows the design of the confocal imaging studies shown in **B**–**G**. As indicated in **A**, we microinstilled fluorescent beads (yellow) in airspaces of 3 imaging fields of at least 30 alveoli in live, perfused lungs of an AT1-CFTR^null^-GFP mouse, then generated images over 2.5 hours to define bead location, group formation, and movement. GFP fluorescence of the imaging fields was in the Medium category. Images in **B** show the initial locations and size distributions of example bead groups that formed within 0.5 hours of beads microinstillation. Dashed squares show locations of the high-power views shown in *i* and *ii*. The arrowhead shows a small cluster of beads located adjacent to a GFP-expressing (cyan) flat alveolar septum, while the arrow points out a microaggregate located at a TdTomato-expressing (magenta) alveolar niche. *mv,* microvessel; *air,* airspace. Images and group data in **C**–**G** show spontaneous movement of all bead groups (**C**–**E**) or small clusters (**F** and **G**) in the 3 imaging fields over the first 3 hours after beads microinstillation. Bead groups were categorized according to whether the adjacent *AT1* cell expressed *WT* CFTR (signaled by TdTomato fluorescence) or *CFTR^null^* (signaled by GFP fluorescence), as indicated. Note in the images in **C** and **F**, beads located adjacent to WT CFTR-expressing AT1 cells moved (**C** and *i* in **F**), but beads adjacent to *CFTR^null^*-expressing AT1 cells did not (*ii* in **F**). In **D** and **G**, circles indicate *n* and each represent 1 bead group. Data are shown as mean ± SEM; *P < 0.05 by 2-tailed *t* test. In **E**, stacked bars represent bead groups as proportions of the total number; *P < 0.05 by χ^2^ test. Scale bars: 30 (**B**), 3 (*i* and *ii* in **B** and **C**), and 15 (**F**) μm.

AWL secretion defends against particle stabilization in alveoli by generating pleura-to-airway liquid flow along alveolar walls that convectively transports particles toward the airways (5). We considered that AWL secretion specifically by AT1 cells might contribute to particle clearance mechanisms. To test this possibility, we used an established method (5, 30) in which we microinstilled fluorescent polystyrene beads (1 μm diameter) into airspaces of Medium-GFP regions of AT1-CFTR^null^-GFP mice (Figure 5A). We analyzed the initial organization and movement of 80 bead groups by generating confocal images of the beads from 30 minutes to 3 hours after microinstillation, followed by measuring their displacement according to: (A) their initial location, defined as bead position against a flat alveolar septum or at an alveolar niche; and (B) their initial group size, defined as a small cluster (1-3 beads) or a microaggregate (4 or more beads).

Our findings show the beads formed small clusters and microaggregates at flat septa and niches within minutes of microinstillation (Figure 5B and Supplemental Figure 5, A and B). Across imaged regions of alveoli, there was no difference in the number of bead groups that initially localized to alveolar walls lined by TdTomato- or GFP-expressing AT1 cells (Supplemental Figure 5A). The distribution of bead subgroups — that is, small clusters at flat septa, microaggregates at flat septa, small clusters at niches, and microaggregates at niches — was also equivalent (Supplemental Figure 5B). Although mean bead group size at TdTomato-expressing AT1 cells was greater than the mean size of bead groups at GFP-expressing AT1 cells (Supplemental Figure 5, C and D), the difference seemed to be driven by a handful of large microaggregates (Supplemental Figure 5E). We interpret that AT1 cell CFTR^null^ expression did not have a major effect on how microinstilled beads organized against alveolar walls.

We next analyzed the movement of beads that were initially located at TdTomato-expressing AT1 cells which, we interpret, expressed WT CFTR. Taking all bead groups together, we found the mean distance of bead displacement was 4 μm along alveolar walls during the imaging period (Figure 5, C–E). However, the distance of bead displacement was not uniform across bead subgroups. The subgroup that moved the least was microaggregates at niches (Supplemental Figure 5F). Bead subgroups that were made up of small clusters or that were located at flat septa moved short distances (Figure 5, F and G, and Supplemental Figure 5G), and the movement was either toward the airways or lateral. No bead groups moved toward the pleura. Together, these data largely support findings published by others that show the liquid flow generated by AWL secretion along alveolar walls transports beads in alveoli away from the pleura and toward the airways (5). In addition, our findings affirm our published data that show microaggregates at alveolar niches are stable in alveoli and resist alveolar clearance mechanisms (30).

We then considered the movement of beads that were located at GFP-expressing AT1 cells which, we interpret, expressed CFTR^null^. As different from beads located at TdTomato-expressing AT1 cells, beads at GFP-expressing AT1 cells were largely immobile (Figure 5, D and E), indicating that AT1 cell CFTR^null^ expression blocked bead movement. Bead immobilization at CFTR^null^-expressing AT1 cells was not caused by microenvironmental factors unrelated to CFTR^null^ expression, since in single alveoli lined on opposite walls by TdTomato- and GFP-expressing AT1 cells, only those beads located at TdTomato-expressing AT1 cells were displaced (Figure 5F). Like niche microaggregates at TdTomato-expressing AT1 cells, niche microaggregates at GFP-expressing AT1 cells moved little, but their relative immobilization was not different from other bead subgroups located at GFP-expressing AT1 cells (Supplemental Figure 5F). Notably, bead subgroups made up of small clusters or located at flat septa lined by GFP-expressing AT1 cells were almost totally immobile, and the distances of their displacements were markedly less than those of their counterparts at TdTomato-expressing AT1 cells (Figure 5, F and G, and Supplemental Figure 5G). These findings show AT1 cell CFTR^null^ expression blocked the displacement of small bead clusters in alveoli, particularly at flat septal locations. We suggest that AT1 cell CFTR contributes to alveolar defense against inhaled particles.

### AT1 cell CFTR promotes S. aureus clearance in alveoli.

**Figure 6:**
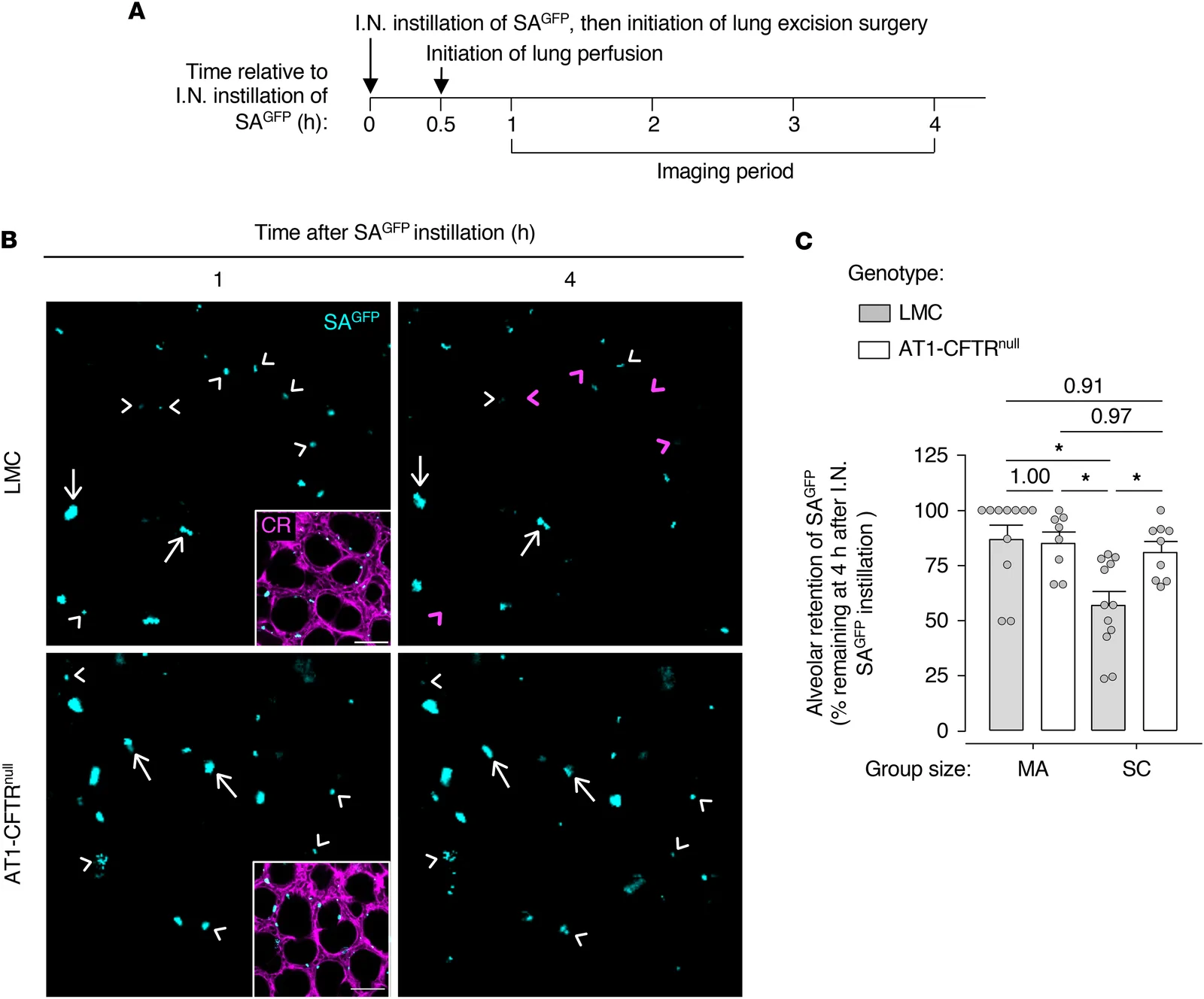
CFTR in AT1 cells promotes bacterial clearance in alveoli. (**A**–**C**) The illustration in **A** shows the experimental design of the confocal imaging studies of bacterial clearance shown in **B** and **C**. As indicated in **A**, mice were given intranasal instillations of SA^GFP^, then the lungs were excised for live imaging 10 minutes later. Confocal images (**B**) and group data (**C**) show microaggregates (arrows; MA) and small clusters (arrowheads; SC) of GFP-expressing *S*. *aureus* (SA^GFP^) in live alveoli of intact, perfused lungs of LMC or AT1-CFTR^null^ mice as indicated. Calcein red-orange (CR) dye was added to the perfusate solution to delineate alveolar walls (insets). Images included at least 30 alveoli and were generated in the same location at 1 and 4 hours after instillation. In the 4-hour images, magenta arrows and arrowheads point out the initial locations of microaggregates and small clusters that were displaced during the imaging period or were not found in the 4-hour images. White arrows and arrowheads in the 4 -hour images indicate microaggregates and small clusters that did not change. In **C**, circles indicate *n* and each represent the change of all small clusters or microaggregates present in one imaging field from 1 to 4 hours after i.n. SA^GFP^ instillation. Imaging fields were generated in the lungs of 3 LMC and 3 AT1-CFTR^null^ mice. Since imaging fields had to be excluded from the analysis if no small clusters or microaggregates were present in the 1-hour image, the data are presented on a per-imaging-field basis rather than a per-mouse basis. Data are shown as mean ± SEM; *P < 0.05 by ANOVA with post hoc Tukey testing. Scale bar: 50 μm.

Since AT1 cell CFTR contributed to alveolar defense against particles, we considered it might also contribute to alveolar defense against inhaled bacteria. To test this possibility, we intranasally instilled mice with GFP-expressing *S*. *aureus* (SA^GFP^) and then imaged the SA^GFP^ in alveoli of live, perfused lungs from 1 to 4 hours after instillation (Figure 6A). We chose this timeframe because our published data show CD11b-expressing neutrophils and monocytes are absent from alveolar airspaces of SA-instilled lungs in this time period (6), reducing the possibility that innate immune responses would confound SA^GFP^ clearance measures. To avoid fluorescence overlap, we instilled SA^GFP^ in nonfluorescent transgenic mice (AT1-CFTR^null^) that carried *Ager-CreER^T2^* and *Cftr^fl10/fl10^* but not *ROSA^mT/mG^*. Littermate control (LMC) mice carried *Cftr^fl10/fl10^*.

Confocal images of live alveoli of AT1-CFTR^null^ and LMC mice at 1 hour after SA^GFP^ instillation show inhaled SA^GFP^ organized against alveolar walls as small clusters of roughly 1–3 bacteria and microaggregates of 4 or more bacteria (Figure 6B). Microaggregates of SA^GFP^ were resistant to alveolar clearance in both AT1-CFTR^null^ and LMC mice (Figure 6, B and C) and, thus, unaffected by AT1 cell CFTR^null^ expression. However, AT1 cell CFTR^null^ expression blocked the clearance of small clusters of SA^GFP^ (Figure 6, B and C). These findings show AT1 cell CFTR contributes to the alveolar clearance of small bacterial clusters. We interpret that loss of AT1 cell CFTR caused retention of inhaled bacteria against alveolar walls, prolonging contact between small bacterial clusters and the alveolar epithelium.

## Discussion

Our findings reveal roles for AT1 cells in lung homeostasis by providing evidence that AT1 cell CFTR contributes to alveolar liquid secretion and the alveolar clearance of particles and inhaled *S*. *aureus*. Specifically, we identified that CFTR^null^ expression in AT1 and AT2 cells each blocked homeostatic AWL secretion, and that AT1 cell CFTR^null^ expression disrupted the alveolar clearance of small clusters of particles and bacteria. Since AT1 cells comprise over 95% of the lung’s air-facing surface (39), we interpret that AT1 cell CFTR is important to lung health and defense. These findings raise the possibility that alveoli, including AT1 cells, are a site of the CFTR dysfunction that underlies CFTR-related lung diseases.

Our identification that AT1 cell CFTR has a role in AWL secretion builds on reports by others that suggest AT1 cells make important contributions to ion and water transport in the lung. For example, Folkesson et al. identified water channels in AT1 cells of sheep lungs and proposed that a major role of the AT1 cells is to transport water (14). Dobbs et al. showed freshly isolated AT1 cells are highly water permeable and suggested AT1 cells account for the high water permeability of the lung (15). Johnson et al. showed freshly isolated rat AT1 cells transport Na^+^ and Cl^–^ and express functional CFTR and ENaC proteins, and the authors proposed that, by virtue of their very large surface area, AT1 cells make major contributions to alveolar fluid balance (16, 18, 40). Our data support these proposals by providing evidence that AT1 cells are drivers of liquid transport in alveoli. Specifically, our finding that AT1 cell CFTR^null^ expression blocked AWL secretion in live alveoli of intact, perfused lungs demonstrates a role for AT1 cells and AT1 cell CFTR in liquid transport under conditions that approximate the structural and physiological complexity of alveoli in vivo. Hence, our findings join a cadre of reports by other groups that suggest AT1 cells are important to liquid and ion transport in the lungs. Since the published reports use data generated in fixed lung tissue and isolated AT1 cells, the advance represented by our study stems from our application of genetic approaches and intact, perfused lungs to show an AT1 cell-specific role for CFTR in homeostatic liquid secretion in alveoli in situ.

Interestingly, the extent of the inhibition of AWL secretion we observed in AT1-CFTR^null^-GFP mice matched the inhibition we observed in AT2-CFTR^null^-GFP and AT2-AT1-CFTR^null^-GFP mice. The equivalent inhibitory effect of CFTR^null^ expression in each of AT1 and AT2 cells shows that both AT1 and AT2 cells make major contributions to homeostatic AWL secretion. Why the inhibition in mice in which either AT1 or AT2 cells expressed CFTR^null^ was not less than the inhibition in mice in which both AT1 and AT2 cells expressed CFTR^null^ is not clear. One possibility is there was ectopic Cre expression in the *Ager-CreER^T2^*– or *Sftpc-CreER^T2^*–expressing mice, such that some AT2 cells expressed CFTR^null^ in AT1-CFTR^null^-GFP mice, or some AT1 cells expressed CFTR^null^ in AT2-CFTR^null^-GFP mice. This might exaggerate the inhibitory effect of CFTR^null^ expression in AT1-CFTR^null^-GFP and AT2-CFTR^null^-GFP mice. However, we do not consider ectopic Cre expression likely to be a major factor in our studies since 2 experiments — first, live lung imaging using the *ROSA^mT/mG^* allele, and second, qPCR of *Cftr* RNA — provide evidence that CFTR^null^ expression in AT1-CFTR^null^-GFP and AT2-CFTR^null^-GFP mice was limited to the intended cell types. Another possibility is that CFTR^null^ expression simultaneously in AT1 and AT2 cells of AT2-AT1-CFTR^null^-GFP mice stimulated a compensatory response in the alveolar epithelium, such as increased expression of non-CFTR Cl^–^ channels like SLC26A9 (41). This might attenuate the inhibitory effect of simultaneous CFTR^null^ expression in AT1 and AT2 cells of AT2-AT1-CFTR^null^-GFP mice, thereby preventing complete inhibition of AWL secretion. Although these questions remain unanswered, we can conclude from our findings that CFTR in both AT1 and AT2 contributes to AWL secretion.

This study adds understanding of alveolar liquid transport mechanisms in lungs at homeostasis. The literature is not clear as to whether alveoli secrete or absorb liquid under homeostatic conditions. For example, data generated by live imaging of fluorophore-labeled dextran and Na^+^ and Cl^–^ indicator dyes in alveolar airspaces of intact, perfused lungs provide evidence that alveoli secrete Na^+^, Cl^–^, and liquid (5, 6, 11). By contrast, data generated by large airway instillation of radionuclides, fluorophore-tagged albumin, Evan’s blue, and other tracers in whole lungs provide evidence that the distal lung epithelium is absorptive (20, 21, 24, 25, 38, 42–52). The reason for the discrepancy between the data generated by alveolar imaging and the data generated by whole lung approaches is not clear. One possibility is the reported liquid absorption in whole lung tissue includes liquid absorption by the airway epithelium, since the whole lung tracer data do not differentiate between airway and alveolar contributions to liquid transport. In line with this possibility, Martens and Ballard report that pig intrapulmonary bronchi secrete liquid when they are air filled, but they absorb liquid when they are fluid filled (53). Notably, the same report indicates that the rate of liquid secretion by air-filled intrapulmonary bronchi is 1.42 ± 0.36 μL•cm^–2^•h^–1^ (53), which, if extrapolated across the 2,471 cm^2^ of airway surface area in human lungs (8), would produce roughly 84 mL of liquid per day — a volume that is meaningfully smaller than that lost daily to respiration (54, 55). If the alveolar epithelium were absorptive under homeostatic conditions, the demand for airway liquid secretion to maintain the lung’s epithelial lining layer would yet increase. Taken in context of the published literature, our findings support the idea that the alveolar epithelium secretes liquid under homeostatic conditions. In addition, our data add evidence that alveolar liquid secretion in live alveoli of perfused lungs is dependent on CFTR in AT1 and AT2 cells.

Our data identify that AT1 cell CFTR displaces small particle clusters from alveolar walls, particularly at flat septa. The mean distance of bead displacement was a modest 4 μm over 2.5 hours of live imaging, and the small distance calls into question the potential biological significance of the displacement. Since we started imaging bead movement 30 minutes after the beads were microinstilled, we might have missed an early period of particle displacement, if particle-epithelial contact stimulates rapid liquid secretion in alveoli as bacteria-epithelial contact does in the airway (56). However, we note that the minimum distance of particle displacement needed to protect against particle-induced epithelial damage in alveoli is not known. Since particle-induced damage to AT2 cells in vitro associates with longer particle exposure times (57), AT1 cell functions like the modest particle displacement we identified could be protective by simply blocking particle stabilization against the alveolar epithelium or resisting particle-epithelial contact. Such resistance to particle contact might be important at flat septa, where the AWL is thin (10) and could bring inhaled particles and AT1 cells close together. Histological data that show CFTR expression on flat surfaces of human alveoli (12, 13) provide support for the idea that flat septa are physiologically significant sites of CFTR function. Keeping these issues in mind, we conclude from our findings that CFTR-dependent liquid secretion by AT1 cells defends against particle stabilization in alveoli by displacing small bead clusters from flat segments of alveolar walls.

Our data also show AT1 cell CFTR contributes to alveolar defense against inhaled bacteria, though the magnitude of the contribution may again be modest. We identified that CFTR^null^ expression in AT1 cells reduced the alveolar clearance of small clusters of inhaled SA^GFP^, indicating that AT1 cell CFTR contributes to small cluster clearance in alveoli. As for particles, the potential biological significance of small bacterial cluster displacement is difficult to know. Since just minutes of contact between epithelial cells and *S*. *aureus* is sufficient to stimulate proinflammatory responses in vitro (58), displacement of small clusters of bacteria from alveolar walls may protect against bacteria-induced alveolar damage and lung inflammation. CFTR-dependent AWL secretion might offer additional epithelial protection by diluting secreted bacterial toxins such as α-hemolysin, which we have shown previously causes epithelial damage in alveoli (30). Hence, the modest bacterial clearance we attribute in this report to AT1 cell CFTR may serve a protective role in alveoli after bacterial inhalation.

The mechanism by which AT1 cell CFTR promoted bacterial clearance is not yet clear. One possibility is the liquid flow generated by AWL secretion directly cleared *S*. *aureus* by transporting the bacteria toward the airways, in the manner proposed by others for particle clearance (5). Another possibility is the secreted liquid indirectly contributed to bacterial clearance by promoting the antibacterial functions of alveolar macrophages, epithelium-derived antimicrobial peptides, and surfactant. Notably, AT1 cell CFTR^null^ expression had no effect on the alveolar clearance of SA^GFP^ that formed microaggregates at alveolar niches. These findings align with our previous reports indicating that microaggregation promotes bacterial stabilization in alveoli (30). The fate of the microaggregated bacteria is not clear, but they might be cleared by other innate immune mechanisms such as neutrophil- or macrophage-mediated bacterial killing. We conclude from our data that AT1 cell CFTR promoted the clearance of small bacterial clusters from alveoli, perhaps contributing to alveolar defense against bacteria-induced epithelial damage. Loss of this defense mechanism in lungs with AT1 cell CFTR dysfunction might prolong bacterial contact with AT1 cells and set the stage for lung infection and lung injury.

Our findings suggest therapies that are intended to promote or restore lung liquid secretion should consider targeting AT1 cells, in addition to other cell types. AT1 cell targeting might be achieved by delivering CFTR modulator drugs or expression vectors in preparations that bind AT1 cell surface proteins, like AGER (29) and EMP2 (59). Our data suggest that effective AT1-targeted inhaled therapies might be designed to not only deposit in alveoli (60, 61), but also to stabilize at flat septa, where we identified a role for AT1 cell CFTR in particle clearance. Although CFTR dysfunction in AT1 cells is not a known feature of CFTR-related lung diseases like CF and COPD, we note that 7 groups have identified that lung alveoli in people with CF harbor tissue remodeling, acute inflammation, and bacteria (62–68), and data generated in lungs of humans with COPD and nonhuman primates with smoke exposure show evidence of alveolar damage (69–72). If shown to be effective, AT1-targeted therapies for CFTR-related diseases have the potential to have an outsized effect by augmenting liquid secretion in a cell type with extensive surface area.

Our study has limitations. First, our findings were generated in mouse lungs, and mice differ from humans in the expression of some ion transport proteins (73). Future studies in human, nonhuman primate, pig, or ferret lungs are needed to define the extent to which our findings are broadly applicable. Second, we relied on male mice in our experiments because we achieved little GFP expression in lungs of female AT1-CFTR^null^-GFP mice, making it challenging to define the effect of AT1 cell CFTR^null^ expression in females. Sex-related differences in responses to tamoxifen (74), which is required to induce CFTR^null^ expression in the transgenic mice used in this study, might underlie the low Cre recombination rates we observed in female mice. Follow-up studies that compare the effects of cell-specific CFTR^null^ expression on alveolar liquid transport in male and female mice might illuminate whether differences in CFTR in AT1 and AT2 cells help explain sex disparities in CFTR-related diseases like CF (75). Data included in a separate report by our group provide evidence that alveoli of female mice secrete AWL at the same rate as males (36), indicating at least some aspects of alveolar liquid transport are preserved in female mice. Third, our studies do not identify the fate of secreted AWL. Publications by others raise the possibility that liquid secreted in alveoli moves onto small airway surfaces, where it might be, to some extent, absorbed (5, 76–78). Our observation that no beads moved toward the pleura in alveoli of LMC-TdTomato mice support the possibility that secreted AWL moves proximally, perhaps due to bulk flow as previously suggested (5). Finally, our experiments do not take into account physical forces of respiration that are applied to alveoli in vivo, and that may influence alveolar fluid distribution and particle transport. Since hyperinflation-induced alveolar expansion affects flat segments of alveoli more than alveolar corners (79), respiration-related physical forces might affect the particle transport we identified, particularly at flat alveolar septa. Dedicated follow-up studies might shed light on these important issues. Despite its limitations, this study provides the first evidence, to our knowledge, that AT1 cell CFTR contributes to liquid transport in live alveoli of perfused lungs.

In conclusion, our findings suggest a shift in the understanding of the role of AT1 cells in lung health and disease. Our data reveal that AT1 cells, traditionally considered to be passive conduits for gas exchange, have important roles in homeostatic alveolar liquid secretion and alveolar defense, and they raise the possibility that loss of AT1 cell CFTR function contributes to CFTR-related lung diseases. Going forward, we suggest that new therapies that aim to augment lung liquid secretion consider AT1 cells as potential therapeutic targets.

## Methods

### Sex as a biological variable.

Our study examined male mice because male animals exhibited more extensive GFP fluorescence in the alveolar epithelium by live lung imaging. It is unknown whether the findings are relevant for female mice.

### Experimental design.

The experiments were designed, and the manuscript was written according to ARRIVE guidelines. Sample sizes and outcome measures are indicated in figures and legends.

### Fluorophores.

We purchased LysoTracker Green (100 nM), Alexa Fluor 647–conjugated dextran (AF-dextran; 10 kDa), FITC-conjugated dextran (FITC-dextran; 20 kDa; 5 mg/mL), and calcein red-orange AM (10 μM) from Thermo Fisher Scientific.

### Reagents.

All reagents were freshly constituted for experiments. We purchased HEPES and Ca^2+^- and Mg^2+^-containing PBS from Thermo Fisher and NaCl, KCl, glucose, CaCl_2_, MgCl_2_, and sucrose from Sigma. Tamoxifen was purchased from Sigma, reconstituted at 20 mg/mL in sunflower oil (Sigma), and administered within 30 minutes of reconstitution. Fluorescent microspheres (1.00 μm, Flash Red, FSFR004) were purchased from Bangs Laboratories Inc.

### Solutions.

Calculated and measured values for solution contents are detailed in Supplemental Table 3 and compared with reported normal values for mouse blood pH (80), plasma osmolality (81), and plasma Na^+^, K^+^, Cl^–^, ionized Ca^2+^, and nonfasting glucose (82–84). We prepared HEPES-buffered solution to serve as the vehicle for the lung perfusate and alveolar instillations. A pH meter (Mettler Toledo Seven Excellence) and NaOH were used to titrate the solution pH. Osmolality was measured by freezing point depression using a micro-osmometer (Precision Systems 6002 Touch Micro Osmette). To generate the lung perfusate solution, we modified the HEPES-buffered vehicle solution by adding dextran (70 kDa; Molecular Probes; 4% w/v) and FBS (Gemini Bio-Products; 1% w/v). FITC-dextran (5 mg/mL) was added to the lung perfusate solution to generate the perfusate solution used for alveolar barrier determinations.

### Bacterial strain and preparation.

*S*. *aureus* was GFP-expressing USA300 LAC (SA^GFP^). SA^GFP^ were stored at –80°C in 25% glycerol in autoclaved Luria-Bertani (LB) broth media (MP Biomedicals) and propagated on LB-agar plates containing chloramphenicol (10 μg/mL). Plates were refreshed from frozen stock every 2 weeks. Single colonies of SA^GFP^ were propagated in autoclaved LB media containing chloramphenicol (10 μg/mL) in a shaking incubator at 37°C and 200 rpm (New Brunswick Scientific) for 18 hours (stationary growth phase). Then, the bacterial solution was prepared for intranasal instillation by diluting 1.3 mL in 200 μL of PBS containing Ca^2+^ and Mg^2+^.

### Animals.

Wild type mice were C57BL/6J (Charles River), and transgenic mice were on a C57BL/6J background. Transgenic strains include: tamoxifen-inducible, *Sftpc-CreER^T2^* (26) (Jackson Laboratory strain #028054); tamoxifen-inducible, *Ager-CreER^T2^* (29) (Jackson Laboratory strain #032771); *Cftr^fl10^* (27) (Case Western Reserve University strain *Cftr^tm1Cwr^*); and *ROSA^mT/mG^* (28) (Jackson Laboratory strain #007676). *Cre* was transmitted through female mice, as recommended by the Case Western Reserve University Cystic Fibrosis Mouse Resource Center. Tail biopsy was performed within 2 weeks of age and sent to Transnetyx for genotyping. All transgenic mice were genotyped. To induce *Cre* expression, we gave intraperitoneal injections of 100 μL of tamoxifen solution in sunflower oil at 8 weeks of age. Mice used for experiments were 16–18 weeks old for AWL studies, 18–24 weeks for PCR studies, and 28–31 weeks old for beads and bacterial studies.

### Intranasal instillation.

SA^GFP^ were prepared for intranasal instillation and instilled within 10 minutes of bacterial removal from the incubator. Mice were anesthetized with inhaled isoflurane (4%) and i.p. injections of ketamine (up to 1 mg) and xylazine (up to 0.1 mg). Each mouse was instilled with 30 μL of SA^GFP^-containing solution to deliver 1 × 10^8^ CFU per mouse. Instillation quality was recorded at the time of instillation by the performing investigator and considered acceptable for experiments if no loss of instillate was observed. Mice woke from anesthesia within 3 minutes of instillation. Within 10 minutes of intranasal instillation, mice were reanesthetized with inhaled isoflurane (4%) and i.p. injections of ketamine (up to 100 mg/kg) and xylazine (up to 5 mg/kg) for euthanasia and surgical preparation of isolated, perfused lungs.

### Preparation of isolated, perfused lungs for imaging.

We anesthetized mice with inhaled isoflurane (4%) and i.p. injections of ketamine (up to 100 mg/kg) and xylazine (up to 5 mg/kg), then gave intracardiac injections of heparin (50 units; Mylan) and exsanguinated the mice by cardiac puncture. Using our reported methods (6, 30), we cannulated the trachea, pulmonary artery, and left atrium of the heart, then excised the heart, lungs, and cannulas en bloc. The lungs were positioned to enable micropuncture and imaging of the diaphragmatic surface of the right middle lobe, right caudal lobe, or left lung. Then, the lungs were inflated with room air through the tracheal cannula and perfused through the pulmonary arterial and left atrial cannulas at 0.5–1.0 mL/minutes with autologous blood diluted in the lung perfusate solution (Supplemental Table 3) and warmed to 37°C. We used in-line pressure transducers (ADInstruments) to maintain constant airway pressure 6 cm H_2_O via a continuous positive airway pressure machine (Philips Respironics) and pulmonary artery and left atrial pressures 10 and 3 cm H_2_O, respectively, via a roller pump (Ismatec). Portions of the lung surface that were not used for micropuncture and imaging were covered with plastic wrap to prevent desiccation.

### Alveolar microinstillation.

We hand-beveled glass micropipettes (Sutter Instruments) to micropuncture single alveoli under bright-field microscopy, as we have done previously (6, 30). Micropunctured alveoli were instilled with fluorophores and reagents in solution, resulting in their spread from the micropunctured alveolus to neighboring alveoli. Microinstillations were performed in 1-3 alveoli bordering each imaging field.

### Live lung imaging and analysis.

By our established methods (6, 30), we viewed alveoli by confocal microscopy (LSM800; Zeiss) with a 20× water immersion objective (NA 1.0; Zeiss) and coverslip. We used bright-field microscopy to randomly select regions of 30–50 alveoli for microinstillation and imaging. All images were acquired as single images using Zen (v.2.6; Zeiss) and recorded as Z-sections. Analyzed images were 4–8 μm below the pleura. Optical thickness was 1 μm for beads imaging and 2 μm for all other experiments, and frame size was 512 × 512 pixels. We established laser, filter, pinhole, and detector settings at the beginning of each imaging experiment to optimize alveolar fluorescence and avoid fluorescence saturation, then maintained the settings for the duration of the experiment. We confirmed absence of bleed-through between fluorescence emission channels. Images were analyzed using ImageJ (NIH; v.2.0.0-rc-69/1.52n). Linear adjustments of brightness and contrast were applied to individual color channels of entire images and equally to all experiment groups. We did not apply downstream processing or averaging.

### Determinations of alveolar liquid transport.

We used alveolar micropuncture to microinstill alveolar airspaces with AF-dextran in HEPES-buffered vehicle solution as described above and Supplemental Table 1. Provided alveolar barrier function is intact, time-dependent loss of fluorophore-tagged dextran fluorescence intensity signals dextran dilution by secreted AWL, while time-dependent increase of dextran fluorescence intensity signals dextran concentration by airspace liquid absorption (5, 6). To measure dextran fluorescence changes, we used ImageJ software (NIH; v.2.0.0-rc-69/1.52n) to quantify AF-dextran gray levels in alveolar airspaces at 2 and 30 minutes after dextran microinstillation. To account for minor heterogeneity of AF-dextran fluorescence changes across imaged alveolar regions, AF-dextran gray levels were quantified as a mean of dextran fluorescence in representative alveoli located at the image top left, top right, bottom left, bottom right, and center. Measurement locations were the same across time points. All experiments were followed by determinations of alveolar barrier permeability. We excluded imaged regions of alveoli from the AF-dextran analysis if: initial AF-dextran gray levels were less than 20; TdTomato fluorescence intensity increased by more than 10% during the imaging period; and in the rare event that permeability determinations showed leak of microvascular fluid into alveolar airspaces.

### Determinations of alveolar barrier permeability.

To determine alveolar barrier properties, we added FITC-dextran (20 kDa; 5 mg/mL) to the intact lung perfusate solution using our established methods (6, 30). FITC-dextran was circulated in the perfusate for 10 minutes prior to image acquisition. Details of the FITC-dextran-containing perfusate solution are included above and Supplemental Table 3.

### Beads quantifications.

We microcentrifuged 1 mL of fluorescent microspheres (“beads”, FSFR004; Bangs Laboratories Inc.) and resuspended them in 20 μL of HEPES-buffered vehicle solution. Beads in HEPES-buffered solution were microinstilled into alveolar airspaces by alveolar micropuncture under bright field microscopy, and then we used confocal microscopy to generate image Z-stacks from 30 minutes to 3 hours after instillation. *ROSA^mT/mG^* fluorescence delineated alveolar walls. To determine bead group size, we used ImageJ (NIH; v.2.0.0-rc-69/1.52n) software to measure the 2-dimensional area of bead groups in single Z-stack images, selecting the Z-stack image in which the bead group was the largest. To define bead group displacement, we used ImageJ to measure the distance from the location of the center of the beads group on the 30 minutes image to the location of the center of the beads group on the 3-hour image. Lateral displacement was defined as the distance (μm) moved between Z-section images that were generated at the same subpleural depth; vertical displacement was defined as the distance (μm) moved between Z-section images generated at different depths, with each Z-section image set at 1 μm apart; and total displacement was the sum of the lateral and vertical displacements.

### Bacterial quantification.

We added calcein red-orange (10 μM) to the lung perfusate solution for 30 minutes, then replaced the perfusate with fluorophore-free perfusate solution. Within 1 hour of intranasal SA^GFP^ instillation, we identified live alveoli with SA^GFP^ and generated confocal images as Z-sections. Additional Z-sections were generated in the same locations at 4 hours after instillation, and then we compared SA^GFP^ fluorescence in the 1- and 4-hour postinstillation images at 6 μM below the pleura. We restricted the analysis of SA^GFP^ to alveoli in which airspace diameters were stable from 1–4 hours after instillation. SA^GFP^ were considered to be retained in alveoli if, on the 4-hour postinstillation image, they were visible on the same Z-section and located within 5 μM of their original location on the 1-hour postinstillation image. SA^GFP^ that merged with calcein fluorescence were considered to be intracellular and excluded from the analysis.

### Mouse lung epithelial cell preparation and fluorescence-activated cell sorting (FACS) of AT2 cells from mouse lungs.

We applied our standard methods (34, 35) to isolate AT2 cells from lung homogenate. Single cell suspension of mouse lungs was created as previously described (85). Briefly, lungs of euthanized mice were instilled with 1 mL of 15 U/mL Dispase II (Thermo Fisher) and incubated with additional 2 mL/mouse of Dispase for 45 minutes at room temperature with agitation. Single-cell suspensions were prepared from digested lungs by incubating with DNAse I (10 minutes at 37°C) and red blood lysis buffer (2 minutes at room temperature) and filtration through 100 μm, 70 μm, and 40 μm filters. For FACS analysis, single-cell preparations were incubated with the following antibodies for 45 minutes at 4°C in DMEM without phenol red + 2% FBS: rat anti-mouse CD45 (1:200 dilution, BD Biosciences, clone 30-F11), rat anti-mouse CD31 (1:200 dilution; BioLegend, clone 390, 102434), rat anti-mouse EpCAM (1:500 dilution, BioLegend, clone G8.8), and rat anti-mouse integrin β4 (1:75 dilution, BD Biosciences, clone 346-11A). Cells were then washed twice with 1× PBS and flash-frozen for RNA isolation. Sorting and subsequent analyses were performed using BD FACS Aria cytometers and FlowJo software.

### qPCR of Cftr RNA.

RNA was extracted from cells using PicoPure RNA Isolation Kit (Applied Biosystems, KIT0204). cDNA was synthesized from total RNA using SuperScript Strand Synthesis System (Thermo Fisher, 18080044). qPCR was performed using SYBR Green (Thermo Fisher, F415L). Relative gene expression levels were defined using the ΔΔCt method. qPCR primers were acquired from Integrated DNA Technologies and are listed in Supplemental Table 2.

### Statistics.

Statistics are indicated in figures and legends. In general, paired comparisons were analyzed using 2-tailed *t* tests, categorical data were analyzed using chi-square testing, and multiple comparisons were analyzed using 1-way ANOVA with post hoc testing. We considered statistical significance at *P* < 0.05. Data were analyzed and figures were prepared using Microsoft Excel, StatPlus:mac Pro (AnalystSoft Inc., Build 7.5.0.0/Core v7.6.11), and SigmaPlot (Systat, version 14.5).

### Study approval.

The IACUC of the Icahn School of Medicine at Mount Sinai approved the animal procedures.

### Data availability.

All data are available in the manuscript, supplemental materials, or Supporting Data Values file.

## Author contributions

JLH designed the study and was responsible for the overall project. JLH, SS, SH, DC, ST, SKLM, and JJK contributed to data collection and data analysis. SS, SH, DC, ST, SKLM, CS, JZ, CJB, AP, AJM, JJK, and JLH contributed to the experimental design and interpretation of results. JLH wrote the first draft and all authors edited the manuscript. The order of the co–first authors was determined based on the greater contribution by SS to manuscript formatting and editing.

## Conflict of interest

The authors have declared that no conflict of interest exists.

## Funding support

This work is the result of NIH funding, in whole or in part, and is subject to the NIH Public Access Policy. Through acceptance of this federal funding, the NIH has been given a right to make the work publicly available in PubMed Central.

- NIH grant R01HL164821 to JLH.
- Cystic Fibrosis Foundation Research Grants 004792G222 and 009144G225 to JLH.
- American Lung Association COVID-19 and Emerging Respiratory Viruses Research Award 1031520 to JLH.
- NIH grant R00HL155785 to JJK.
- American Lung Association Hastings Innovation Award HIA-1278383 to JJK.

## Supplementary Material

Supplemental data

Supporting data values

## Version 1 08/11/2026

In-Press Preview

## Version 2 09/22/2026

Electronic publication
