# Supplementary material for: CFTR in alveolar type 1 cells contributes to lung liquid secretion and host defense
Source: JCI Insight. 2026 Aug 11;11(18):e194121. doi: 10.1172/jci.insight.194121 (PMC13596726; doi:10.1172/jci.insight.194121)
Supplement: Supplementary file 1 [file jciinsight-11-194121-s299.pdf]

SUPPLEMENTAL DATA

CFTR IN ALVEOLAR TYPE 1 CELLS CONTRIBUTES TO LUNG LIQUID SECRETION AND HOST DEFENSE

Sayahi Suthakaran, Sonya Homami, Deebly Chavez, Stephanie Tang, Sarah K. L. Moore, Chaya Sussman, Jimmy Zhang, Clemente J. Britto, Alice Prince, Alison J. May, Jaymin J. Kathiriya, and Jaime L. Hook

Supplemental Figure 1

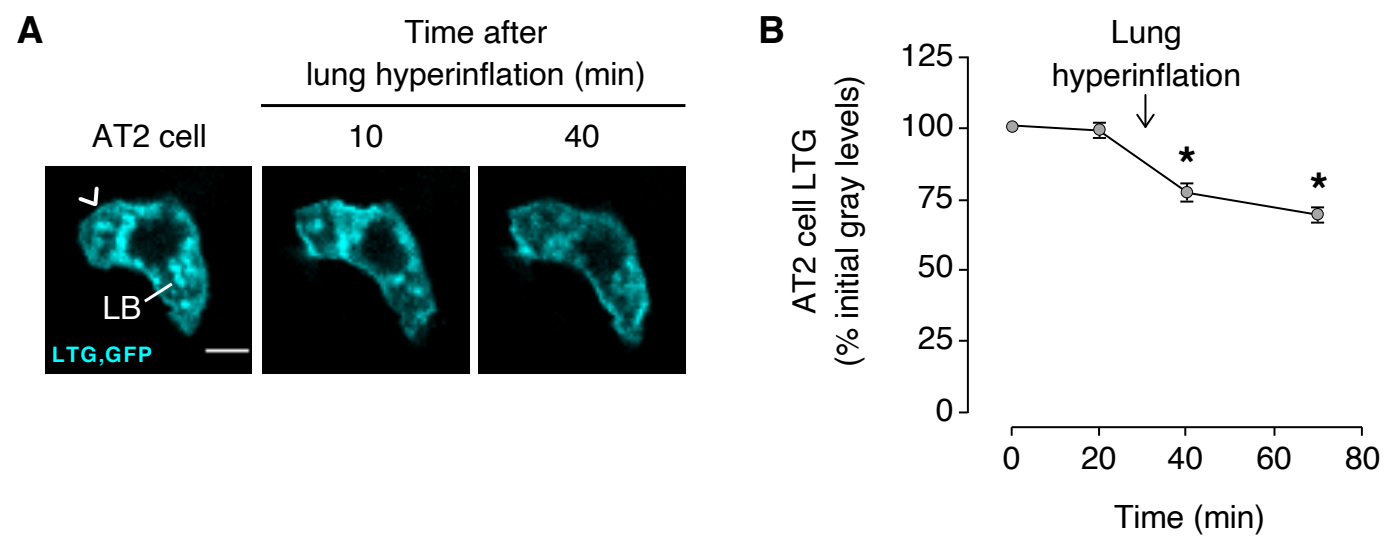

Supplemental Figure 1. Functional phenotyping of AT2 cells in transgenic mice. (**A-B**) Confocal images (A) show fluorescence of AT2 cell lamellar bodies (*LBs*) loaded with lysotracker green (*LTG*) before (*left*) and after (*middle* and *right*) a 15-second lung hyperinflation from 6 to 15 cm H_2O in an AT2-CFTR^{null}-GFP mouse. Plasma membrane *GFP* fluorescence (*arrowhead*) confirms *Sftpc* expression. Note, LTG fluorescence loss indicates hyperinflation-induced surfactant secretion and affirms AT2 cell identity. Both LTG and GFP are pseudocolored cyan due to fluorescence overlap. In B, *circles* indicate *n* and each represent mean ± SEM of 3 trials of LTG microinstillation in which mean LTG fluorescence was quantified in imaging fields of at least 30 alveoli. *$p < 0.05$ versus the initial value as indicated by ANOVA with post hoc Tukey testing. Scale bar: 5 μm.

Supplemental Figure 2

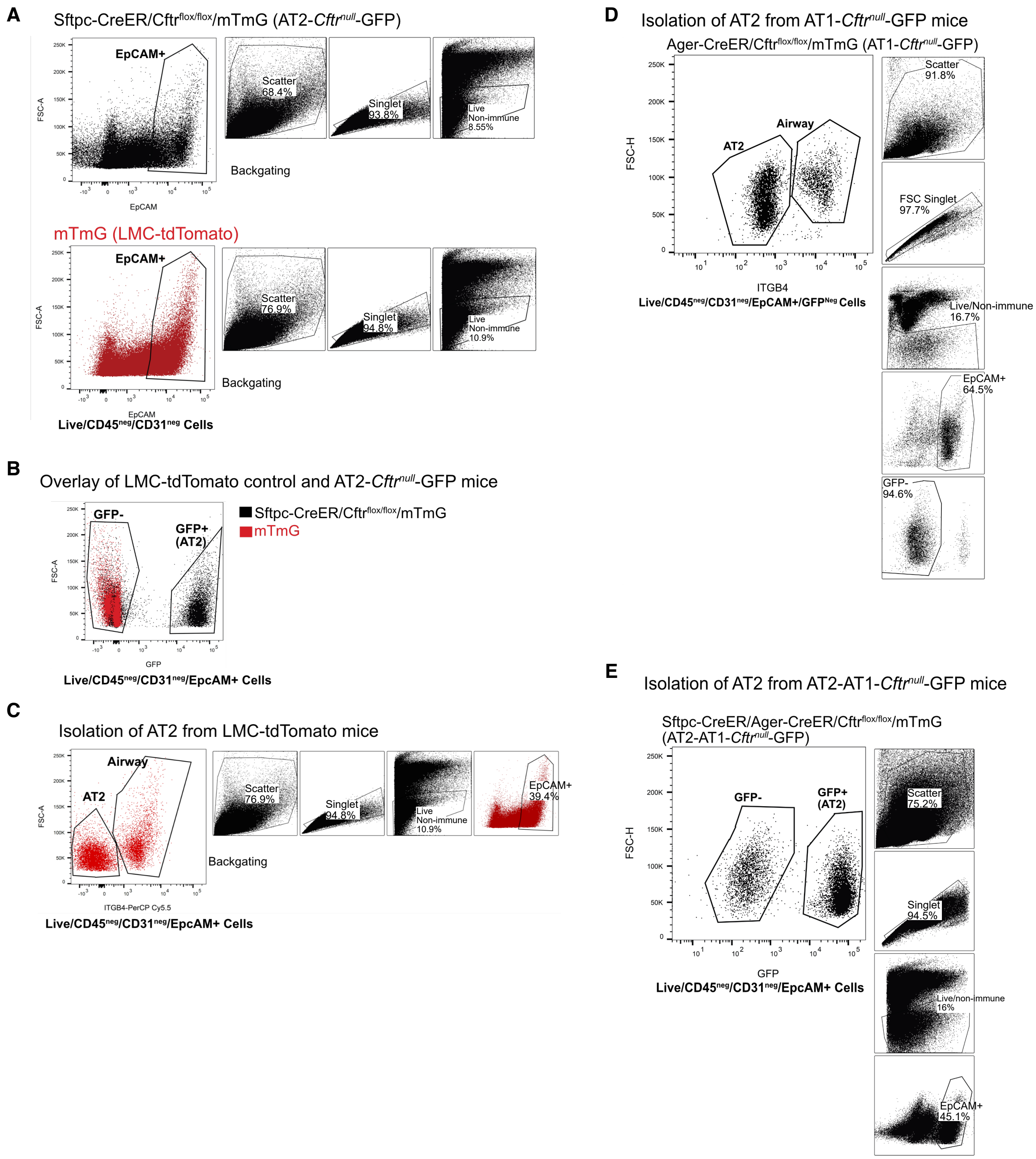

Supplemental Figure 2. Flow sorting strategy of AT2 cells from lung homogenate. **(A)** Flow plot shows strategy for identifying EpCAM+ cells within Live/CD45^{neg}/CD31^{neg} cells prepared from single cell suspensions from lungs of AT2-CFTR^{null}-GFP (*top panel*) and LMC-TdTomato mice (*bottom panel*). Backgating shows similar levels of live/non-immune cells. **(B)** Overlay of EpCAM+ cells from AT2-CFTR^{null}-GFP and LMC-TdTomato mice against GFP expression shows specificity of tamoxifen-induced GFP expression. GFP+ cells were isolated as AT2 cells from the

AT2-CFTR^{null}-GFP mouse. **(C)** Strategy of isolating AT2 cells from LMC-TdTomato mice where cells were stained for ITGB4 (an airway epithelial marker). EpCAM+/ITGB4^{neg} were isolated as AT2 cells from the LMC-TdTomato mouse. **(D)** GFP^{neg}/EpCAM+/ITGB4^{neg} were sorted as AT2 cells from AT1-CFTR^{null}-GFP mice. Less than 5% of GFP+ cells were observed in backgating, consistent with compromised extraction of AT1 cells using standard lung digestion protocols. **(E)** Isolation of AT2 cells from AT2-AT1-CFTR^{null}-GFP mice. GFP+ cells were sorted as AT2 cells. Experiments were performed using one mouse per genotype of LMC-TdTomato, AT2-CFTR^{null}-GFP, AT1-CFTR^{null}-GFP, and AT2-AT1-CFTR^{null}-GFP mice. All mice were tamoxifen-treated.

Supplemental Figure 3

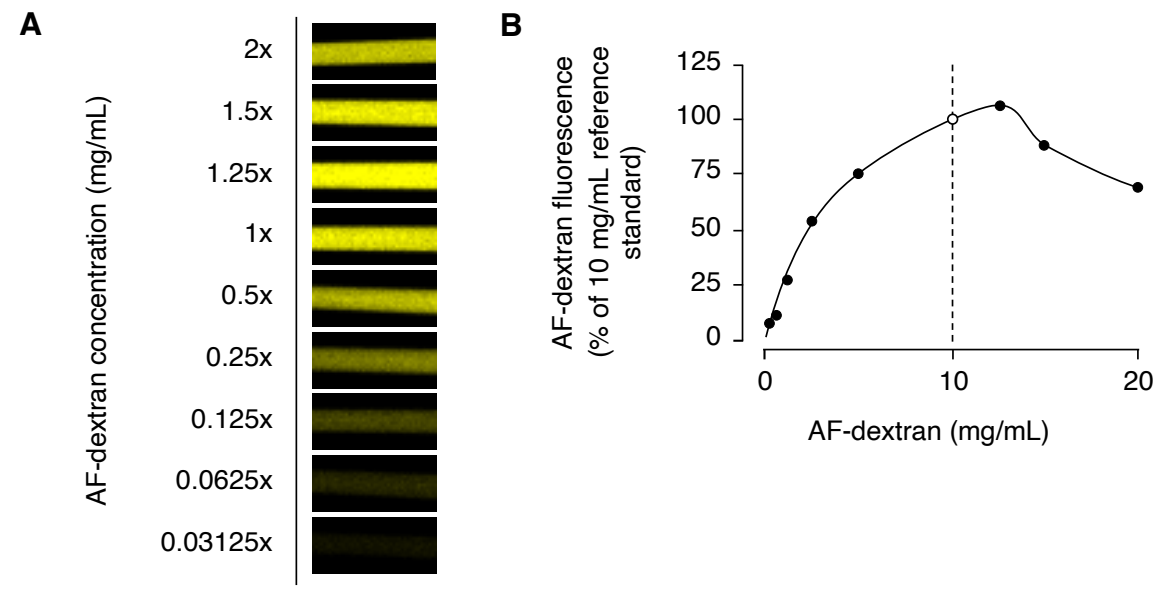

Supplemental Figure 3. Fluorescence of AF-dextran in glass micropipettes. (A-B) Confocal images (A) and plot (B) show the relationship between Alexa Fluor 647-conjugated dextran (*AF-dextran,* 10 kDa) concentration and fluorescence intensity in aqueous solution in glass micropipettes. The *open circle* and *dashed line* in B indicate the 10 mg/mL reference standard.

Supplemental Figure 4

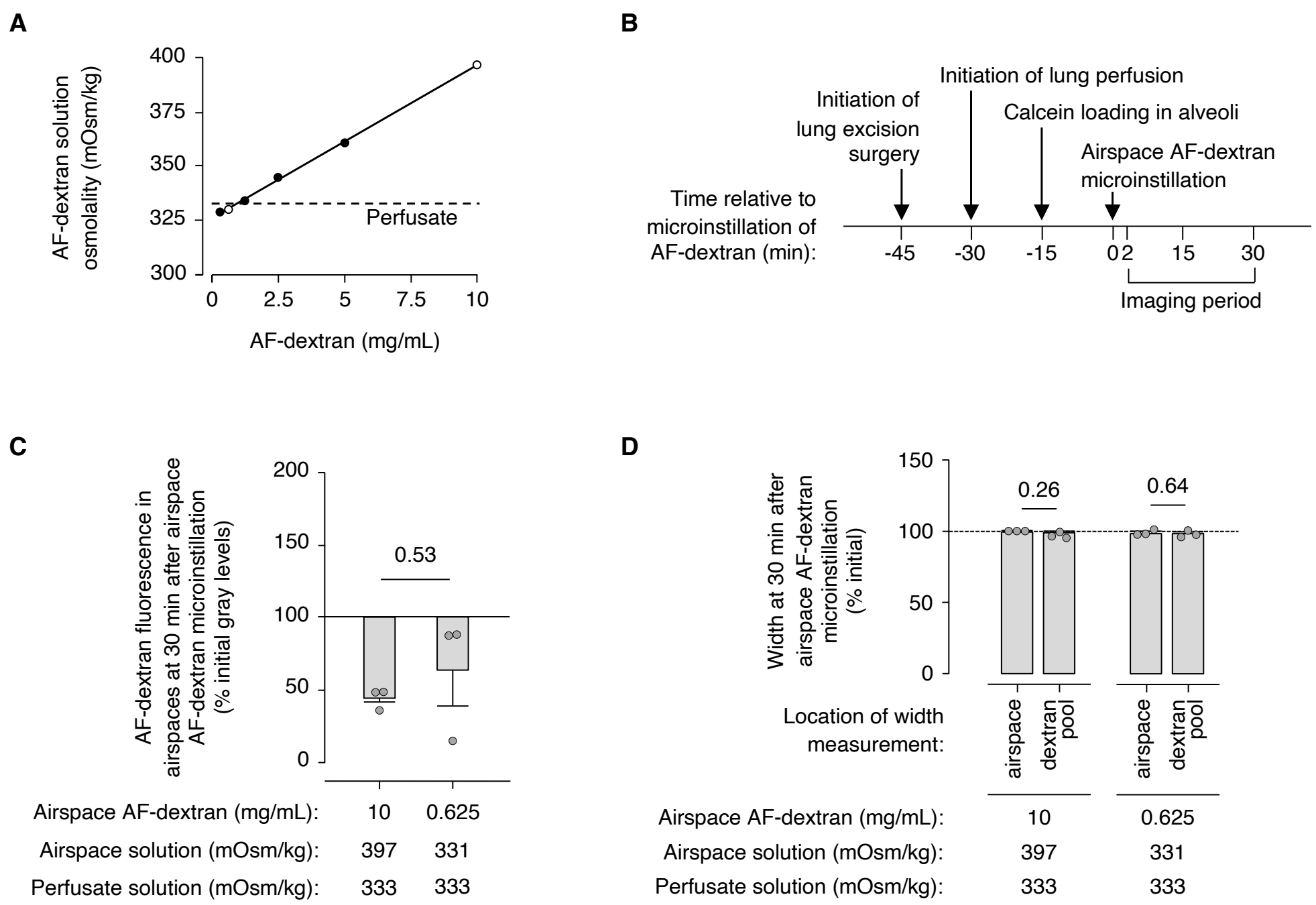

Supplemental Figure 4. Trans-alveolar osmotic gradient does not determine the direction of liquid transport in alveoli. (A-D) The plot in A shows the effect of Alexa Fluor 647-conjugated dextran (*AF-dextran,* 10 kDa) concentration on solution osmolality. *White circles* indicate the AF-dextran-containing solutions that were microinstilled into alveolar airspaces to carry out the experiments shown in B-D. The *dashed line* shows the osmolality of the lung perfusate solution, which did not contain AF-dextran, that was used in the same experiments shown in B-D. The cartoon in B shows the design of experiments used to generate the confocal imaging group data shown in C-D. Group data in C-D show change of airspace AF-dextran fluorescence (C), airspace AF-dextran pool width (D), and airspace width (D) at 30 min after microinstillation of the indicated AF-dextran solutions in live alveoli of perfused lungs. In C-D, *circles* indicate n and each represent one C57BL/6 mouse in which mean change of fluorescence and widths were quantified in imaging fields of at least 30 alveoli; *bars* show mean ± SEM; *p as indicated by two-tailed t test.

Supplemental Figure 5

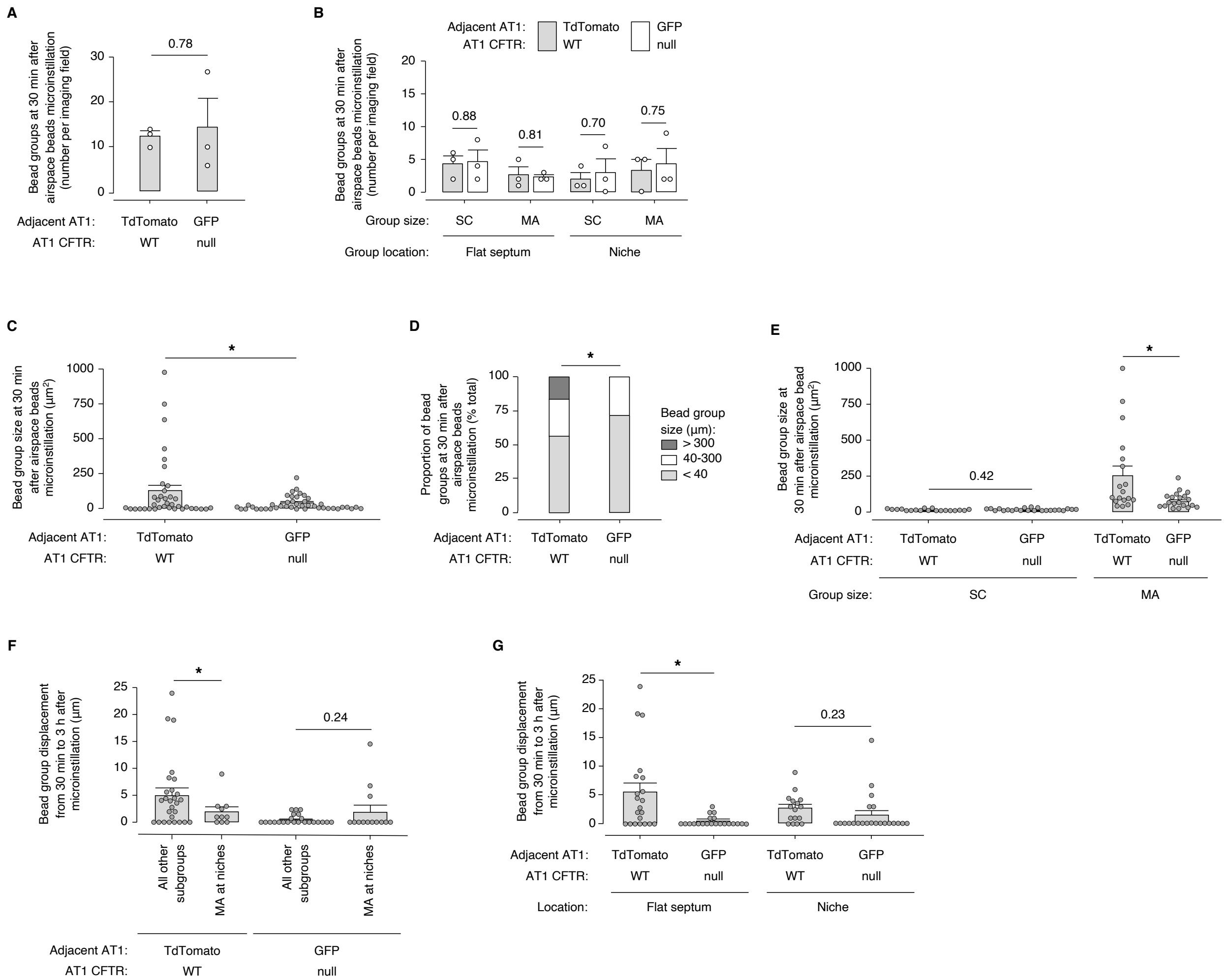

Supplemental Figure 5. Confocal imaging of fluorescent beads in live alveoli of intact, perfused lungs. (**A-G**) Group data are a continuation of the confocal imaging data presented in Figure 5. We microinstilled fluorescent beads in airspaces of 3 imaging fields of at least 30 alveoli in live, perfused lungs of an AT1-CFTR^{null}-GFP mouse, then generated confocal images of the imaging fields for 3 h to define bead group formation and movement. GFP fluorescence of the imaging fields was in the Medium category. The group data in A-G show the initial locations (A-B), initial sizes in two dimensions (C-E), and subsequent displacement distances (F-G) in the 3 imaging fields over the first 3 h after beads microinstillation. Bead groups were classified as small clusters (*SC*) or microaggregates (*MA*), and their initial locations in alveoli were categorized according to whether the *adjacent AT1* cell expressed *WT* CFTR (signaled by TdTomato fluorescence) or *CFTR^{null}* (signaled by GFP fluorescence), as indicated. The group data in C-D include all bead groups, while E divides them by group size. *Circles* indicate n. In A-B, *open circles* each represent the mean number of bead groups in one imaging field, while in C and E-G, *closed circles* each represent one bead group. In A-C and E-G, *bars* indicate mean ± SEM. *Stacked bars* in D represent bead groups as proportions of the total number. *p < 0.05 or as indicated by Chi-squared test (D) or two-tailed t test (all others).

Supplemental Table 1

Genotype	*Cftr* RNA in AT2 cells
LMC-TdTomato	1.00 (reference value)
AT2-$CFTR^{null}$-GFP	< 0.01
AT1-$CFTR^{null}$-GFP	1.27
AT2-AT1-$CFTR^{null}$-GFP	< 0.01

Supplemental Table 1. *Cftr* RNA in AT2 cells of transgenic mice. qPCR results by mouse genotype. At least 50,000 AT2 cells were collected per genotype.

Supplemental Table 2

Name	Sequence
mCftr-F2_e10	GAGAACAATGTCAGTTTCAGTCATC
mCftr-R2_e11	CCCAAAATCAACATCAGGAGTG
L19-F1	ATGTATCACAGCCTGTACCTG
L19-R1	TTCTTGGTCTCTTCCTCCTTG

Supplemental Table 2. qPCR primers. The indicated primers were used for qPCR of *Cftr* RNA.

Supplemental Table 3

	Direct measures							Calculated values			
	pH	osmolality (mOsm/kg)	Na^+ (mM)	K^+ (mM)	Cl^- (mM)	ionized Ca^{2+} (mM)	glucose (mg/dL)	Mg^{2+} (mM)	HEPES (mM)	D70 (% w/v)	FBS (% w/v)
Normal blood and plasma values in mice	7.4	323-330	146-151	3.8-4.4	107-111	1.10-1.13	164-176				
Lung perfusate solution	7.4	333	153	4.9	143	1.09	171	1.0	20	4	1
HEPES-buffered vehicle solution for instillations	7.4	324	151	4.8	143	1.00	168	1.0	20	0	0
plus AF-dextran at 10 mg/mL		397									
plus AF-dextran at 0.625 mg/mL		331									

Supplemental Table 3. Composition of airspace and lung perfusate solutions. Ionized Ca^{2+} values are corrected for pH. *D70*, dextran (70 kDa); *FBS*, fetal bovine serum. Citations for normal values are indicated in the Methods.
